# Supplementary material for: Efficacy of combined COVID-19 convalescent plasma with oral RNA-dependent RNA polymerase inhibitor treatment versus neutralizing monoclonal antibody therapy in COVID-19 outpatients: a multi-center, non-inferiority, open-label randomized controlled trial (PlasMab)
Source: Microbiol Spectr. 2023 Nov 17;11(6):e03257-23. doi: 10.1128/spectrum.03257-23 (PMC10714803; doi:10.1128/spectrum.03257-23)
Supplement: Supplemental file 1 — Supplemental material. [file spectrum.03257-23-s0001.pdf]

Supplementary

Inclusion criteria

1. Age equal or more than 20 years

2. Body weigh equal or more than 40 kilograms

3. SARS-CoV-2 detected from nasopharyngeal specimen by antigen test kit and confirmed with SARS-CoV-2 PCR

4. Symptomatic equal or less than 7 days after onset of a symptom: At least one of the following symptom; fever, cough, runny nose, sore throat, dyspnea, chest discomfort, diarrhea

5. Absence or presence <50%of pneumonia from chest X-ray or presence of pneumonia from chest CT with overall CT severity score 1-14 (mild to moderate pneumonia)

6. WHO clinical progression scale < /= 3

7. Thai language communicable

8. Accept to follow up according to outpatient treatment or home isolation protocol

9. At least one of the following risk factor to deteriorate to severe illness: age more than 60 years, obesity or BMI >/= 30 kg/ m2, diabetes mellitus, chronic kidney disease, heart disease and cardiovascular disease, COPD/ asthma, hypertension, liver disease with child pugh score A-B, receiving immunosuppressive therapy, cerebrovascular accident/stroke, intellectual disabilities, cancer with life expectancy more than 3 months, patients who disabilities and need medical intervention include tracheostomy, gastrostomy

10. Accept the informed consent

Exclusion criteria

1. Pregnancy or breastfeeding

2. Severe illness: including pneumonia with the following condition

2.1 Respiratory rates more than 24 per minute

2.2 Oxygen saturation less than 95%

3. Need for oxygen therapy

4. chronic oxygen therapy with the need to increase oxygen flow from the other pulmonary disease except from COVID-19

5. Patient who need hospitalization

6. Chest X-ray showed more than 50% bilateral infiltration, or chest CT showed multifocal consolidation, crazy paving pattern, ARDS

7. Intake the other anti-SARS-CoV-2 therapy more than 24 hours

8. Child-Pugh score C or end-stage-renal disease without dialysis or hemodialysis less than two times/week

9. Advanced stage cancer or palliative treatment (with life expectancy less than three months)

10. Bed-ridden and need hospitalization

11. Received neutralizing monoclonal antibody or plasma in 60 days

12. severe allergic reaction to favipiravir or sotrovimab

13. History of severe transfusion reaction

Table S1 Baseline Characteristics

Parameters	Sotrovimab (n=68)	COVID-19 Convalescent Plasma/ Favipiravir (n=68)	P-value
Sex, n (%)			1.000[1]
Male	29 (42.65)	29 (42.65)	
Female	39 (57.35)	39 (59.35)	
Age, mean ± SD	64.34 ± 1.49	61.97 ± 1.79	0.312[3]
Elderly	95 (69.85)	45 (66.18)	0.350[1]
Duration from onset of illness, mean ± SD	2.66 ± 1.61	2.62 ± 1.46	0.986[4]
BMI, mean ± SD	25.86 ± 4.67	27.47 ± 7.90	0.451[4]
BMI, n (%)			0.467[2]
<18	1 (1.49)	2 (2.94)	
18-24.9	33 (49.25)	31 (45.59)	
25-29.9	22 (32.84)	18 (26.47)	
≥30	11 (16.42)	17 (25.00)	
Variant (Variant of Concern), n (%)			0.881[2]
Omicron	1 (1.47)	0 (0.00)	
Omicron BA.1	1 (1.47)	1 (1.47)	
Omicron BA.1.1	2 (2.94)	5 (7.35)	
Omicron BA.2	20 (29.41)	19 (27.94)	
Mix	3 (4.41)	2 (2.94)	
Unknown	41 (60.29)	41 (60.29)	
Previous COVID-19 infection, n (%)	2 (3.13)	4 (5.88)	0.681[2]
Vaccination status, n (%)			0.274[2]
Unvaccinated	17 (25.00)	10 (14.71)	
Incomplete vaccination	1 (1.47)	0 (0.00)	
Fully inactivated or ChAdOx-1 nCoV vaccination	12 (17.65)	13 (19.12)	
Third-dose heterologous vaccination	29 (42.65)	39 (57.35)	
Fourth dose with at least 1 dose mRNA	9 (13.24)	6 (8.82)	
Type of vaccination, n (%)			0.541[2]
- Unvaccinated	17 (25.00)	10 (14.71)	
- Coronavac-Coronavac	1 (1.47)	0 (0.00)	
- BBIBP-CorV-BBIBP-CorV	2 (2.94)	2 (2.94)	
- ChAdOx-1 nCoV-ChAdOx-1 nCoV	7 (10.29)	10 (14.71)	
- BNT162b2-BNT162b2	1 (1.47)	1 (1.47)	

- ChAdOx-1 nCoV-BNT162b2	1 (1.47)	0 (0.00)	
- Coronavac-Coronavac-ChAdOx-1 nCoV	4 (5.88)	3 (4.41)	
- Coronavac-Coronavac-mRNA-1273	1 (1.47)	1 (1.47)	
- Coronavac-Coronavac-BNT162b2	1 (1.47)	1 (1.47)	
- Coronavac-ChAdOx-1 nCoV-ChAdOx-1 nCoV	1 (1.47)	0 (0.00)	
- ChAdOx-1 nCoV-ChAdOx-1 nCoV-ChAdOx-1 nCoV	0 (0.00)	5 (7.35)	
- ChAdOx-1 nCoV-ChAdOx-1 nCoV-mRNA-1273	2 (2.94)	1 (1.47)	
- ChAdOx-1 nCoV-ChAdOx-1 nCoV-BNT162b2	17 (25.00)	23 (33.82)	
- BBIBP-CorV-BBIBP-CorV-BNT162b2	2 (2.94)	1 (1.47)	
- BBIBP-CorV-BBIBP-CorV-ChAdOx-1 nCoV	0 (0.00)	2 (2.94)	
- BBIBP-CorV-ChAdOx-1 nCoV-BNT162b2	0 (0.00)	1 (1.47)	
- mRNA-1273-mRNA-1273-BNT162b2	1 (1.47)	0 (0.00)	
- Coronavac-Coronavac-ChAdOx-1 nCoV-ChAdOx-1 nCoV	1 (1.47)	1 (1.47)	
- Coronavac-Coronavac-ChAdOx-1 nCoV-mRNA-1273	1 (1.47)	0 (0.00)	
- Coronavac-Coronavac-ChAdOx-1 nCoV-BNT162b2	5 (7.35)	4 (5.88)	
- Coronavac-Coronavac-BNT162b2-BNT162b2	0 (0.00)	1 (1.47)	
- BBIBP-CorV-BBIBP-CorV-ChAdOx-1 nCoV-ChAdOx-1 nCoV	1 (1.47)	0 (0.00)	
- ChAdOx-1 nCoV-ChAdOx-1 nCoV-mRNA-1273-Unknown	1 (1.47)	0 (0.00)	
- Unknown vaccination	1 (1.47)	0 (0.00)	
- Unknown-Unknown-Unkonwn vaccination	0 (0.00)	1 (1.47)	
Comorbidities, n (%)			
Obesity	11 (16.42)	18 (26.47)	0.155[1]
Malignancy	7 (10.29)	6 (8.82)	0.771[1]
Diabetes mellitus	15 (22.06)	17 (25.00)	0.686[1]
Hypertension	29 (42.65)	42 (61.76)	0.026[1]
Chronic Kidney Disease	3 (4.41)	1 (1.47)	0.619[2]
Coronary artery disease	4 (5.88)	1 (1.47)	0.366[2]
Chronic lung disease	1 (1.47)	1 (1.47)	1.000[2]
Asthma	1 (1.47)	1 (1.47)	0.119[2]
Heart Disease, n (%)	5 (7.35)	6 (8.82)	1.000[1]
Cerebrovascular disease	3 (4.41)	5 (7.35)	0.718[2]
HIV infection	1 (1.47)	0 (0.00)	1.000[2]
Reapiratory rate (/minute)	19.80 ± 1.26	19.58 ± 1.52	0.331[4]
Body temperature	36.42 ± 0.27	36.53 ± 0.42	0.185[4]
Oxygen saturation	98.72 ± 1.28	98.70 ± 1.30	0.955[4]
Symptoms, n (%)			
- Cough	47 (69.12)	48 (72.73)	0.646[1]
- Fever	12 (17.65)	14 (21.21)	0.602[1]

- Sore throat	27 (39.71)	30 (45.45)	0.501[1]
- Runny nose	25 (36.76)	33 (50.00)	0.122[1]
- Myalgia	5 (7.35)	7 (10.61)	0.510[1]
- Dyspnea	2 (2.94)	1 (1.52)	1.000[2]
- Chest tightness	3 (4.41)	1 (1.54)	0.620[2]
- Diarrhea	3 (4.41)	1 (1.52)	0.619[2]
Mild illness	46 (67.65)	47 (69.12)	0.854[1]
Pneumonia	22 (32.35)	21 (30.88)	
Ct value of SARS-CoV-2 PCR ORF-1 a/b gene, mean ± SD	20.08 ± 3.18	20.82 ± 4.73	0.958[4]
Positive anti-spike RBD antibodies level > 1000	61.00 (89.71)	54.00 (79.41)	0.097[1]
White blood cell Day 0	6.02 ± 1.67	5.84 ± 1.93	0.223[4]
Absolute Lymphocyte Day 0	1.70 ± 0.64	1.85 ± 0.52	0.083[4]
Platelet Day 0	233.94 ± 61.67	232.10 ± 55.34	0.918[4]
Hemoglobin Day 0	14.67 ± 12.66	13.12 ± 1.63	0.593[4]
HbA1C Day 0	6.14 ± 1.45	6.33 ± 1.67	0.876[4]
Blood sugar Day 0	111.83 ± 46.15	121.83 ± 53.45	0.372[4]
BUN Day 0	13.31 ± 5.45	13.05 ± 3.96	0.800[4]
Creatinine Day 0	0.97 ± 0.64	0.88 ± 0.27	0.190[4]
AST Day 0	25.72 ± 9.52	26.82 ± 11.85	0.307[4]
ALT Day 0	23.61 ± 15.61	25.25 ± 18.28	0.381[4]
ALP Day 0	75.55 ± 19.68	72.31 ± 18.25	0.625[4]
hsCRP	13.97 ± 17.17	8.86 ± 10.18	0.080[4]
Presepsin	967.58 ± 456.64	971.25 ± 432.00	0.703[4]
D-dimer	638.26 ± 1,177.81	389.60 ± 357.95	0.891[4]
LDH	196.22 ± 37.59	185.31 ± 32.58	0.031[4]
Ferritin	320.88 ± 242.10	326.29 ± 305.30	0.523[4]
Procalcitonin	0.12 ± 0.29	0.08 ± 0.09	0.441[4]
ESR	46.97 ± 23.51	56.24 ± 23.47	0.824[4]

Abbreviations; n: number; Ct: cycle threshold; BMI: Body Mass Index; PCR: polymerase Chain Reaction; SD: Standard deviation; RBD: receptor-binding domain; SARS-CoV 2: severe acute respiratory syndrome coronavirus-2; COVID-19: Coronavirus disease 2019; ESR: Erythrocyte sedimentation rate; LDH: lactate dehydrogenase; hsCRP: high-sensitive C-reactive protein:AST: Aspatate transaminase; ALT: Alanine aminotransferase; ALP: Alkaline phosphatase; BUN: blood urea nitrogen; HIV: Human Immunodeficiency Virus

[1]Pearson Chi-square test [2]Fisher's exact test [3]Independent t-test [4]Mann-Whitney U test

Table S2 Clinical symptoms change after COVID-19 convalescent plasma therapy and favipiravir versus sotrovimab treatment

Parameters	Sotrovimab (n=68)	COVID-19 Convalescent Plasma/ Favipiravir (n=68)	P-value
Cough on Day 0 , n (%)	47 (69.12)	48 (72.73)	0.646[1]
- Presence	47 (69.12)	48 (72.73)	
- Absence	21 (30.88)	18 (27.27)	
Cough on Day 2, n (%)			0.865[2]
- Absence	17 (25.37)	14 (21.54)	
- Stable	22 (32.84)	20 (30.77)	
- Worsening	1 (1.49)	2 (3.08)	
- Improvement	27 (40.30)	29 (44.62)	
Cough on Day 5, n (%)			0.061[2]
- Absence	18 (26.87)	10 (14.93)	
- Stable	6 (8.96)	14 (20.90)	
- Worsening	4 (5.97)	1 (1.49)	
- Improvement	39 (58.21)	42 (62.69)	
Cough on Day 14, n (%)			0.320[2]
- Absence	18 (26.47)	12 (18.18)	
- Stable	2 (2.94)	0 (0.00)	
- Worsening	1 (1.47)	2 (3.03)	
- Improvement	47 (69.12)	52 (78.79)	
Dyspnea on Day 0, n (%)	2 (2.94)	1 (1.52)	1.000[2]
- Presence	2 (2.94)	1 (1.52)	
- Absence	66 (97.06)	65 (98.48)	
Dyspnea on Day 2, n (%)			0.744[2]
- Absence	66 (98.51)	63 (96.92)	
- Stable	1 (1.49)	1 (1.54)	
- Worsening	0 (0.00)	0 (0.00)	
- Improvement	0 (0.00)	1 (1.54)	
Dyspnea on Day 5, n (%)			1.000[2]
- Absence	65 (97.01)	65 (97.01)	
- Stable	0 (0.00)	1 (1.49)	
- Worsening	0 (0.00)	0 (0.00)	
- Improvement	2 (2.99)	1 (1.49)	
Dyspnea Day 14, n (%)			0.559[2]
- Absence	64 (95.52)	62 (93.94)	

Parameters	Sotrovimab (n=68)	COVID-19 Convalescent Plasma/ Favipiravir (n=68)	P-value
- Stable	1 (1.49)	0 (0.00)	
- Worsening	0 (0.00)	0 (0.00)	
- Improvement	2 (2.99)	4 (6.06)	
Chest discomfort on Day 0, n (%)	3 (4.41)	1 (1.54)	0.620[2]
- Presence	3 (4.41)	1 (1.54)	
- Absence	65 (95.59)	64 (98.46)	
Chest discomfort on Day 2, n (%)			0.180[2]
- Absence	63 (94.03)	65 (100.00)	
- Stable	1 (1.49)	0 (0.00)	
- Worsening	0 (0.00)	0 (0.00)	
- Improvement	3 (4.48)	0 (0.00)	
Chest discomfort on Day 5, n (%)			0.244[2]
- Absence	64 (95.52)	67 (100.00)	
- Stable	0 (0.00)	0 (0.00)	
- Worsening	0 (0.00)	0 (0.00)	
- Improvement	3 (4.48)	0 (0.00)	
Chest discomfort on Day 14, n (%)			0.244[2]
- Absence	64 (95.52)	66 (100.00)	
- Stable	0 (0.00)	0 (0.00)	
- Worsening	0 (0.00)	0 (0.00)	
- Improvement	3 (4.48)	0 (0.00)	
Fever on Day 0, n (%)	12 (17.65)	14 (21.21)	0.602[1]
- Presence	12 (17.65)	14 (21.21)	
- Absence	56 (82.35)	52 (78.79)	
Fever on Day 2, n (%)			0.631[2]
- Absence	52 (77.61)	51 (78.46)	
- Stable	5 (7.46)	2 (3.08)	
- Worsening	0 (0.00)	1 (1.54)	
- Improvement	10 (14.93)	11 (16.92)	
Fever on Day 5, n (%)			1.000[2]
- Absence	51 (76.12)	51 (76.12)	
- Stable	2 (2.99)	1 (1.49)	
- Worsening	0 (0.00)	0 (0.00)	
- Improvement	14 (20.90)	15 (22.39)	
Fever on Day 14, n (%)			0.840[2]

Parameters	Sotrovimab (n=68)	COVID-19 Convalescent Plasma/ Favipiravir (n=68)	P-value
- Absence	52 (77.61)	50 (75.76)	
- Stable	0 (0.00)	0 (0.00)	
- Worsening	0 (0.00)	0 (0.00)	
- Improvement	15 (22.39)	16 (24.24)	
Sore throat on Day 0, n (%)	27 (39.71)	30 (45.45)	0.501[1]
- Presence	27 (39.71)	30 (45.45)	
- Absence	41 (60.29)	36 (54.55)	
Sore throat on Day 2, n (%)			0.934[2]
- Absence	41 (61.19)	38 (58.46)	
- Stable	7 (10.45)	8 (12.31)	
- Worsening	0 (0.00)	1 (1.54)	
- Improvement	19 (28.36)	18 (27.69)	
Sore throat on Day 5, n (%)			0.340[2]
- Absence	41 (61.19)	39 (58.21)	
- Stable	0 (0.00)	3 (4.48)	
- Worsening	1 (1.49)	0 (0.00)	
- Improvement	25 (37.31)	25 (37.31)	
Sore throat on Day 14, n (%)			0.535[2]
- Absence	42 (62.69)	38 (57.58)	
- Stable	1 (1.49)	0 (0.00)	
- Worsening	0 (0.00)	0 (0.00)	
- Improvement	24 (35.82)	28 (42.42)	
Runny nose on Day 0, n (%)	25 (36.76)	33 (50.00)	0.122[1]
- Presence	25 (36.76)	33 (50.00)	
- Absence	43 (63.24)	33 (50.00)	
Runny nose on Day 2, n (%)			0.301[2]
- Absence	39 (58.21)	31 (47.69)	
- Stable	11 (16.42)	9 (13.85)	
- Worsening	0 (0.00)	2 (3.08)	
- Improvement	17 (25.37)	23 (35.38)	
Runny nose on Day 5, n (%)			0.358[2]
- Absence	40 (59.70)	31 (46.27)	
- Stable	2 (2.99)	4 (5.97)	
- Worsening	2 (2.99)	1 (1.49)	
- Improvement	23 (34.33)	31 (46.27)	

Parameters	Sotrovimab (n=68)	COVID-19 Convalescent Plasma/ Favipiravir (n=68)	P-value
Runny nose on Day 14, n (%)			0.410[2]
- Absence	39 (58.21)	31 (46.97)	
- Stable	1 (1.49)	1 (1.52)	
- Worsening	0 (0.00)	0 (0.00)	
- Improvement	27 (40.30)	34 (51.52)	
Diarrhea on Day 0, n (%)	3 (4.41)	1 (1.52)	0.619[2]
- Presence	3 (4.41)	1 (1.52)	
- Absence	65 (95.59)	65 (98.48)	
Diarrhea on Day 2, n (%)			0.838[2]
- Absence	64 (95.52)	62 (95.38)	
- Stable	0 (0.00)	1 (1.54)	
- Worsening	0 (0.00)	0 (0.00)	
- Improvement	3 (4.48)	2 (3.08)	
Diarrhea on Day 5, n (%)			1.000[2]
- Absence	64 (95.52)	65 (97.01)	
- Stable	0 (0.00)	0 (0.00)	
- Worsening	0 (0.00)	0 (0.00)	
- Improvement	3 (4.48)	2 (2.99)	
Diarrhea on Day 14, n (%)			1.000[2]
- Absence	64 (95.52)	64 (96.97)	
- Stable	0 (0.00)	0 (0.00)	
- Worsening	0 (0.00)	0 (0.00)	
- Improvement	3 (4.48)	2 (3.03)	

Abbreviations; n: number; COVID-19: Coronavirus disease 2019
[1]Pearson chi-square test [2]Fisher's exact test [3]Independent t-test [4]Mann-Whitney U test

Table S3 Adverse event of sotrovimab and combined COVID-19 convalescent plasma therapy and favipiravir

**Adverse Events**	**Sotrovimab (n=68)**	**COVID-19 Convalescent Plasma/ Favipiravir (n=68)**	**P-value**
Anaphylaxis	0 (0.00)	2 (3.03)	0.241[2]

Abbreviations; n: number; COVID-19: Coronavirus disease 2019

Table S4 Radiologic outcome of sotrovimab versus combined COVID-19 convalescent plasma therapy with favipiravir

**Parameters**	**Sotrovimab (n=68)**	**COVID-19 Convalescent Plasma/ Favipiravir (n=68)**	**P-value**
CT severity index score at Day 0	0.73 ± 1.41	0.61 ± 1.82	0.336[4]
CT severity index score at Day 5	0.75± 1.37	0.88 ± 1.88	0.823[4]
Pneumonia Diagnosis at Day 0, n (%)			0.854[1]
- Mild illness	46 (67.65)	47 (69.12)	
- Pneumonia	22 (32.35)	21 (30.88)	
Pneumonia Diagnosis at Day 5, n (%)			0.726[1]
- Mild illness	42 (61.76)	40 (58.82)	
- Pneumonia	26 (38.24)	28 (41.18)	
Radiologic chage by chest CT on Day 5, n (%)			0.258[2]
- Stable no pneumonia	42 (61.76)	38 (55.88)	
- Stable pneumonia	22 (32.35)	19 (27.94)	
- Progression	4 (5.88)	9 (13.24)	
- Relsolution	0 (0.00)	2 (2.94)	

Abbreviations; n: number; CT: Computed tomography; COVID-19: Coronavirus disease 2019
[1]Pearson Chi-square test [2]Fisher's exact test [3]Independent t-test [4]Mann-Whitney U

Table S5 Virological outcome of sotrovimab versus combined COVID-19 convalescent plasma therapy with favipiravir

**Parameters**	**Sotrovimab (n=68)**	**COVID-19 Convalescent Plasma/ Favipiravir (n=68)**	**P-value**
PCR E-gene Day 0, mean ± SD	21.03 ± 4.90	20.38 ± 3.41	0.997[4]
PCR E-gene Day 5, mean ± SD	29.01 ± 4.57	29.39 ± 4.63	0.634[3]
PCR E-gene Day 14, mean ± SD	37.34 ± 2.93	27.13 ± 2.86	0.694[4]
PCR ORF-1 Day 0, mean ± SD	20.08 ± 3.18	20.82 ± 4.73	0.958[4]
PCR ORF-1 Day 5, mean ± SD	28.15 ± 3.87	28.16 ± 3.75	0.843[4]
PCR ORF-1 Day 14, mean ± SD	33.08 ± 1.93	33.00 ± 1.69	0.194[4]
Negative PCR Day 5, n (%)	9 (13.43)	5 (7.35)	0.273[1]
Negative PCR Day 14, n (%)	62 (91.18)	57 (83.82)	0.195[1]

Abbreviations; n: number; COVID-19: Coronavirus disease 2019; PCR: Polymerase Chain Reaction
[1]Pearson chi-square test [2]Fisher's exact test [3]Independent t-test [4]Mann-Whitney U test

Table S6 Inflammatory marker changes after sotrovimab versus combined COVID-19 convalescent plasma therapy with favipiravir

Inflammatory markers	Sotrovimab (n=68)	COVID-19 Convalescent Plasma/ Favipiravir (n=68)	P-value
hsCRP			
Day 0	13.97 ± 17.17	8.86 ± 10.18	0.080[4]
Day 2	9.25 ± 10.87	6.60 ± 8.88	0.074[4]
Day 5	3.34 ± 3.28	6.17 ± 12.72	0.927[4]
Day 14	2.64 ± 4.14	2.90 ± 4.42	0.535[4]
Presepsin			
Day 0	967.58 ± 456.64	971.25 ± 432.00	0.703[4]
Day 2	247.66 ± 327.53	191.76 ± 118.40	0.679[4]
Day 5	585.78 ± 292.11	555.98 ± 212.87	0.869[4]
Day 14	579.78 ± 368.82	520.78 ± 228.91	0.598[4]
D-dimer			
Day 0	638.26 ± 1,177.81	389.60 ± 357.95	0.891[4]
Day 2	577.19 ± 816.86	774.74 ± 1,240.30	0.137[4]
Day 5	766.88 ± 1,307.58	437.69 ± 462.77	0.380[4]
Day 14	653.37 ± 1,137.24	477.93 ± 568.00	0.724[4]
LDH			
Day 0	196.22 ± 37.59	185.31 ± 32.58	0.031[4]
Day 2	197.57 ± 40.26	195.54 ± 37.61	0.686[4]
Day 5	196.38 ± 34.22	191.32 ± 40.51	0.108[4]
Day 14	190.57 ± 30.93	182.00 ± 33.55	0.061[4]
Ferritin			
Day 0	320.88 ± 242.10	326.29 ± 305.30	0.523[4]
Day 2	312.52 ± 230.83	339.31 ± 318.65	0.990[4]
Day 5	307.70 ± 225.54	339.71 ± 364.91	0.986[4]
Day 14	248.19 ± 188.06	259.64 ± 239.76	0.761[4]
Procalcitonin			
Day 0	0.12 ± 0.29	0.08 ± 0.09	0.441[4]
Day 2	0.09 ± 0.29	0.09 ± 0.16	0.342[4]
Day 5	0.06 ± 0.09	0.07 ± 0.11	0.442[4]
Day 14	0.06 ± 0.09	0.04 ± 0.02	0.798[4]
ESR			
Day 0	46.97 ± 23.51	56.24 ± 23.47	0.824[4]
Day 5	42.07 ± 22.86	45.16 ± 24.48	0.409[4]

Day 14	37.51 ± 25.97	41.64 ± 25.60	0.252[4]

Abbreviations; n: number; COVID-19: Coronavirus disease 2019; ESR: Erythrocyte sedimentation rate; LDH: lactate dehydrogenase; hsCRP: high-sensitive C-reactive protein

[1]Pearson Chi-square test [2]Fisher's exact test [3]Independent t-test [4]Mann-Whitney U test

Table S7 Biochemical changes after sotrovimab versus combined COVID-19 convalescent plasma therapy with favipiravir

**Parameters**	**Sotrovimab (n=68)**	**COVID-19 Convalescent Plasma/ Favipiravir (n=68)**	**P-value**
AST Day 0	25.72 ± 9.52	26.82 ± 11.85	0.307[4]
AST Day 2	24.77 ± 7.66	26.01 ± 10.75	0.824[4]
AST Day 5	22.42 ± 8.28	22.78 ± 9.60	0.700[4]
AST Day 14	21.88 ± 10.02	21.94 ± 7.45	0.614[4]
ALT Day 0	23.61 ± 15.61	25.25 ± 18.28	0.381[4]
ALT Day 2	22.86 ± 13.78	24.34 ± 17.42	0.570[4]
ALT Day 5	21.46 ± 13.41	23.41 ± 18.49	0.314[4]
ALT Day 14	21.61 ± 18.63	21.08 ± 13.92	0.592[4]
ALP Day 0	75.55 ± 19.68	72.31 ± 18.25	0.625[4]
ALP Day 2	72.87 ± 18.75	72.48 ± 17.29	0.645[4]
ALP Day 5	71.71 ± 20.24	73.31 ± 18.96	0.345[4]
ALP Day 14	73.21 ± 21.48	73.98 ± 21.73	0.675[4]
Uric Day 0	5.35 ± 1.39	5.35 ± 1.39	0.812[4]
Uric Day 5	5.07 ± 1.47	8.48 ± 2.33	<0.001[3]
Uric Day 14	5.35 ± 1.31	5.62 ± 1.40	0.251[3]

Abbreviations; n: number; COVID-19: Coronavirus disease 2019: Aspatate transaminase; ALT: Alanine aminotransferase; ALP: Alkaline phosphatase

[1]Pearson Chi-square test [2]Fisher's exact test [3]Independent t-test [4]Mann-Whitney U test

Table S8 Immunologic response after sotrovimab versus combined COVID-19 convalescent plasma with favipiravir treatment

Parameters	Sotrovimab (n=68)	COVID-19 Convalescent Plasma/ Favipiravir (n=68)	P-value
CLIA SARS-CoV-2 IgM Day 0	0.40 ± 1.04	0.81 ± 3.13	0.508[4]
CLIA SARS-CoV-2 IgM Day 2	0.72 ± 2.91	0.78 ± 2.60	0.769[4]
CLIA SARS-CoV-2 IgM Day 5	1.18 ± 3.66	0.74 ± 2.07	0.353[4]
CLIA SARS-CoV-2 IgM Day 14	1.17 ± 3.40	0.95 ± 3.10	0.608[4]
CLIA SARS-CoV-2 IgM Day 28	0.27 ± 0.37	0.68 ± 2.15	0.703[4]
CLIA SARS-CoV-2 IgM Day 0 positive, n (%)	0.00 (0.00)	1.00 (1.52)	0.493[2]
CLIA SARS-CoV-2 IgM Day 2 positive, n (%)	1.00 (1.47)	1.00 (1.47)	1.000[2]
CLIA SARS-CoV-2 IgM Day 5 positive, n (%)	3.00 (4.55)	1.00 (1.47)	0.362[2]
CLIA SARS-CoV-2 IgM Day 14 positive, n (%)	1.00 (1.49)	1.00 (1.47)	1.000[2]
CLIA SARS-CoV-2 IgM Day 28 positive, n (%)	0.00 (0.00)	1.00 (1.69)	0.492[2]
CLIA SARS-CoV-2 IgG Day 0	5.85 ± 18.29	2.15 ± 8.38	0.426[4]
CLIA SARS-CoV-2 IgG Day 2	8.77 ± 23.68	13.00 ± 22.39	<0.001[4]
CLIA SARS-CoV-2 IgG Day 5	16.80 ± 32.25	18.45 ± 30.49	0.023[4]
CLIA SARS-CoV-2 IgG Day 14	22.61 ± 30.99	19.83 ± 28.39	0.712[4]
CLIA SARS-CoV-2 IgG Day 28	6.84 ± 13.92	5.68 ± 13.90	0.420[4]
CLIA SARS-CoV-2 IgG Day 0 positive, n (%)	4.00 (5.97)	2.00 (3.03)	0.680[2]
CLIA SARS-CoV-2 IgG Day 2 positive, n (%)	11.00 (16.18)	18.00 (26.47)	0.143[1]
CLIA SARS-CoV-2 IgG Day 5 positive, n (%)	14.00 (21.21)	21.00 (30.88)	0.203[1]
CLIA SARS-CoV-2 IgG Day 14 positive, n (%)	21.00 (31.34)	23.00 (33.82)	0.759[1]
CLIA SARS-CoV-2 IgG Day 28 positive, n (%)	3.00 (5.00)	1.00 (1.67)	0.619[2]
Quantitative anti-RBD spike IgG Day 0	11,609.50 ± 16,604.74	10,771.82 ± 14,326.89	0.772[4]
Quantitative anti-RBD spike IgG Day 2	38,771.72 ± 15,418.39	15,219.76 ± 18,029.57	<0.001[4]
Quantitative anti-RBD spike IgG Day 5	37,173.84 ± 16,511.02	24,500.22 ± 20,811.12	<0.001[4]
Quantitative anti-RBD spike IgG Day 14	42,822.00 ± 21,181.78	37,132.67 ± 23,747.10	0.041[4]
Quantitative anti-RBD spike IgG Day 28	43,554.56 ± 23,002.50	37,368.87 ± 24,875.27	0.065[4]
Positive anti-RBD spike IgG Day 0, n (%)	61.00 (89.71)	54.00 (79.41)	0.097[1]
Positive anti-RBD spike IgG Day 2, n (%)	68.00 (100.00)	61.00 (89.71)	0.013[2]
Positive anti-RBD spike IgG Day 5, n (%)	67.00 (100.00)	66.00 (97.06)	0.496[2]
Positive anti-RBD spike IgG Day 14, n (%)	68.00 (100.00)	66.00 (98.51)	0.496[2]
Positive anti-RBD spike IgG Day 28, n (%)	63.00 (100.00)	60.00 (98.36)	0.492[2]
sVNT Day 0	77.51 ± 36.12	86.33 ± 25.78	0.245[4]

sVNT Day 2	86.07 ± 27.80	96.25 ± 12.23	0.208[4]
sVNT Day 5	89.88 ± 25.53	99.20 ± 1.81	0.025[4]
sVNT Day 14	92.51 ± 22.15	98.79 ± 6.09	0.160[4]
sVNT Day 28	93.82 ± 19.25	98.20 ± 9.96	0.265[4]
Positive Neutralizing antibody Day 0, n (%)	57.00 (83.82)	63.00 (92.65)	0.110[1]
Positive Neutralizing antibody Day 2, n (%)	61.00 (89.71)	67.00 (98.53)	0.062[2]
Positive Neutralizing antibody Day 5, n (%)	54.00 (90.00)	61.00 (100.00)	0.013[2]
Positive Neutralizing antibody Day 14, n (%)	62.00 (92.54)	68.00 (100.00)	0.028[2]
Positive Neutralizing antibody Day 28, n (%)	59.00 (93.65)	60.00 (98.36)	0.365[2]

Abbreviations; n: number; RBD: receptor-binding domain; SARS-CoV 2: severe acute respiratory syndrome coronavirus-2; COVID-19: Coronavirus disease 2019; sVNT: surrogated virus neutralizing test; CLIA: chemiluminescent immunoassay

[1]Pearson Chi-square test [2]Fisher's exact test [3]Independent t-test [4]Mann-Whitney U test

Table S9 Cytokine response after sotrovimab versus combined COVID-19 convalescent plasma with favipiravir treatment

Cytokines	Sotrovimab (n=68)	COVID-19 Convalescent Plasma/ Favipiravir (n=68)	P-value
IL-1α Day 0	0.21 ± 0.25	0.24 ± 0.39	0.541[4]
IL-1α Day 2	0.17 ± 0.21	0.27 ± 0.66	0.888[4]
IL-1α Day 5	0.24 ± 0.35	0.27 ± 0.67	0.848[4]
IL-1α Day 14	0.15 ± 0.20	0.16 ± 0.18	0.568[4]
IL-1β Day 0	3.07 ± 9.31	4.05 ± 11.19	0.795[4]
IL-1β Day 2	2.02 ± 5.68	1.82 ± 3.11	0.535[4]
IL-1β Day 5	1.88 ± 4.31	3.69 ± 10.05	0.818[4]
IL-1β Day 14	0.93 ± 0.50	2.19 ± 5.51	0.132[4]
IL-2 Day 0	1.81 ± 3.90	2.14 ± 5.55	0.596[4]
IL-2 Day 2	1.98 ± 4.67	1.87 ± 4.25	0.433[4]
IL-2 Day 5	2.04 ± 5.45	2.58 ± 6.09	0.542[4]
IL-2 Day 14	1.73 ± 5.17	1.41 ± 1.73	0.273[4]
IFN-γ Day 0	0.98 ± 1.02	1.21 ± 1.99	0.920[4]
IFN-γ Day 2	0.48 ± 0.54	0.61 ± 0.97	0.954[4]
IFN-γ Day 5	0.28 ± 0.57	0.35 ± 0.87	0.617[4]
IFN-γ Day 14	0.29 ± 0.48	0.26 ± 0.44	0.883[4]
TNF-α Day 0	12.61 ± 35.57	29.61 ± 114.25	0.219[4]
TNF-α Day 2	7.18 ± 12.81	6.46 ± 5.76	0.238[4]
TNF-α Day 5	8.94 ± 12.69	35.75 ± 117.55	0.284[4]
TNF-α Day 14	4.69 ± 3.97	11.38 ± 22.35	0.231[4]
IL-4 Day 0	1.47 ± 1.74	1.51 ± 1.64	0.575[4]
IL-4 Day 2	1.55 ± 1.94	1.43 ± 1.66	0.749[4]
IL-4 Day 5	1.64 ± 2.36	2.60 ± 5.87	0.865[4]
IL-4 Day 14	1.57 ± 2.06	1.24 ± 0.55	0.951[4]
IL-6 Day 0	25.99 ± 102.06	24.37 ± 104.57	0.271[4]
IL-6 Day 2	12.98 ± 51.61	9.60 ± 37.82	0.582[4]
IL-6 Day 5	20.47 ± 52.28	28.54 ± 106.99	0.211[4]
IL-6 Day 14	2.68 ± 5.04	26.36 ± 106.74	0.279[4]
IL-8 Day 0	166.13 ± 198.84	165.48 ± 199.75	0.569[4]
IL-8 Day 2	120.33 ± 217.63	120.28 ± 198.70	0.795[4]
IL-8 Day 5	137.88 ± 231.42	127.25 ± 220.37	0.521[4]
IL-8 Day 14	85.78 ± 159.00	138.88 ± 205.87	0.201[4]

IL-10 Day 0	1.04 ± 0.81	29.79 ± 135.54	0.018[4]
IL-10 Day 2	1.28 ± 1.48	29.38 ± 135.61	0.129[4]
IL-10 Day 5	0.59 ± 0.48	30.07 ± 138.64	0.316[4]
IL-10 Day 14	0.39 ± 0.29	29.48 ± 138.76	0.650[4]
VEGF Day 0	146.69 ± 85.48	126.04 ± 79.36	0.271[4]
VEGF Day 2	141.16 ± 84.31	123.26 ± 83.88	0.418[4]
VEGF Day 5	166.79 ± 92.34	156.61 ± 112.02	0.378[4]
VEGF Day 14	156.01 ± 88.32	147.31 ± 84.44	0.657[4]
MCP-1 Day 0	200.23 ± 120.89	236.36 ± 142.72	0.401[4]
MCP-1 Day 2	143.13 ± 65.60	174.21 ± 67.28	0.108[3]
MCP-1 Day 5	140.72 ± 65.00	155.57 ± 62.91	0.422[3]
MCP-1 Day 14	130.46 ± 49.88	175.67 ± 80.49	0.026[3]
EGF Day 0	165.52 ± 59.83	161.74 ± 62.98	0.831[3]
EGF Day 2	140.39 ± 53.90	151.35 ± 65.67	0.526[3]
EGF Day 5	144.70 ± 61.31	150.81 ± 65.41	0.738[3]
EGF Day 14	158.55 ± 68.47	158.12 ± 61.33	0.599[4]

Abbreviations: IL, interleukin; IFN, interferon; TNF, tumor necrosis factor; MCP-1, monocyte chemoattractant protein-1; VEGF, vascular endothelial growth factor; EGF: Endothelial Growth Factor
[1]Pearson Chi-square test [2]Fisher’s exact test [3]Independent t-test [4]Mann-Whitney U test

Table S10 Subgroup analysis of sotrovimab versus combined COVID-19 convalescent plasma with favipiravir treatment among unvaccinated and at least 2-dose vaccination

	Unvaccinated			At least 2-dose vaccination		
	Sotrovimab (n=17)	Convalescent Plasma Therapy with Favipiravir (n=10)	P-value	Sotrovimab (n=50)	Convalescent Plasma Therapy with Favipiravir (n=58)	P-value
Clinical on Day 2			0.142[2]			0.902[1]
- Stable	5 (29.41)	0 (0.00)		14 (28.00)	15 (25.86)	
- Worsening	1 (5.88)	1 (10.00)		4 (8.00)	6 (10.34)	
- Improvement	11 (64.71)	9 (90.00)		32 (64.00)	37 (63.79)	
Clinical on Day 5			0.516[2]			0.629[2]
- Stable	0 (0.00)	0 (0.00)		4 (8.00)	2 (3.45)	
- Worsening	2 (11.76)	0 (0.00)		2 (4.00)	3 (5.17)	
- Improvement	15 (88.24)	10 (100.00)		44 (88.00)	53 (91.38)	
Clinical on Day 14			1.000[2]			0.451[2]
- Stable	0 (0.00)	0 (0.00)		0 (0.00)	2 (3.45)	
- Worsening	0 (0.00)	0 (0.00)		1 (2.00)	3 (5.17)	
- Improvement	17 (100.00)	10 (100.00)		49 (98.00)	53 (91.38)	
Chest CT on Day 5, n (%)			1.000[2]			0.844[2]
- No Pneumonia	10 (58.82)	6 (60.00)		31 (62.00)	34 (58.62)	
- Stable infiltration	7 (41.18)	4 (40.00)		19 (38.00)	24 (41.38)	
- Worsening	0 (0.00)	0 (0.00)		0 (0.00)	0 (0.00)	
- Improvement	0 (0.00)	0 (0.00)		0 (0.00)	0 (0.00)	
Pneumonia presented on Day 0, n (%)			0.204[2]			0.785[1]
- Mild illness	11 (64.71)	9 (90.00)		34 (68.00)	38 (65.52)	
- Pneumonia	6 (35.29)	1 (10.00)		16 (32.00)	20 (34.48)	
Pneumonia presented on Day 5, n (%)			1.000[2]			0.721[1]
- Mild illness	10 (58.82)	6 (60.00)		31 (62.00)	34 (58.62)	
- Pneumonia	7 (41.18)	4 (40.00)		19 (38.00)	24 (41.38)	
Negative PCR Day 5, n (%)	2 (12.50)	0 (0.00)	0.508[2]	7 (14.00)	5 (5.62)	0.375[1]
Negative PCR Day 14, n (%)	14 (82.35)	4 (40.00)	0.365[2]	47 (94.00)	51 (87.93)	0.278[1]
WHO-CPS score on Day 0, n (%)			1.000[2]			1.000[2]
- 0	0 (0.00)	0 (0.00)		0 (0.00)	0 (0.00)	
- 1	0 (0.00)	0 (0.00)		0 (0.00)	0 (0.00)	
- 2	17 (100.00)	10 (100.00)		50 (100.00)	58 (100.00)	
WHO-CPS score on Day 5, n (%)			1.000[2]			0.610[2]
- 0	1 (5.88)	0 (0.00)		6 (12.00)	4 (6.90)	
- 1	15 (88.24)	10 (100.00)		38 (76.00)	48 (82.76)	
- 2	1 (5.88)	0 (0.00)		6 (12.00)	6 (10.34)	
WHO-CPS score on Day 14, n (%)			0.335[2]			0.278[2]
- 0	14 (82.35)	6 (60.00)		45 (90.00)	46 (79.31)	
- 1	3 (17.65)	3 (30.00)		5 (10.00)	11 (18.97)	
- 2	0 (0.00)	1 (10.00)		0 (0.00)	1 (1.72)	

Abbreviatioons; WHO-CPS: World Health Organization Clinical Progression Scale; n: number

[1]Pearson chi-square test [2]Fisher's exact test

Table S11 Subgroup analysis of sotrovimab versus combined COVID-19 convalescent plasma with favipiravir treatment among those who presented RBD spike IgG antibody equal/ less than 1000 and more than 1000

	RBD spike IgG antibody ≤ 1000			RBD spike IgG antibody > 1000		
	Sotrovimab	Convalescent Plasma Therapy with Favipiravir	P-value	sotrovimab	Convalescent Plasma Therapy with Favipiravir	P-value
Clinical on Day 2			1.000[2]			1.000[2]
- Stable	13 (100.00)	15 (100.00)		0 (0.00)	0 (0.00)	
- Worsening	0 (0.00)	0 (0.00)		54 (100.00)	54 (100.00)	
- Improvement	0 (0.00)	0 (0.00)		0 (0.00)	0 (0.00)	
Clinical on Day 5			0.087[2]			1.000[2]
- Stable	1 (7.69)	0 (0.00)		3 (5.56)	2 (3.77)	
- Worsening	2 (15.38)	0 (0.00)		2 (3.70)	3 (5.66)	
- Improvement	10 (76.92)	15 (100.00)		49 (90.74)	48 (90.57)	
Clinical on Day 14			1.000[2]			0.332[2]
- Stable	0 (0.00)	0 (0.00)		0 (0.00)	2 (3.77)	
- Worsening	0 (0.00)	1 (6.67)		1 (1.85)	2 (3.77)	
- Improvement	13 (100.00)	14 (93.33)		53 (98.15)	49 (92.45)	
Chest CT on Day 5, n (%)			1..000[2]			0.499[2]
- No Pneumonia	6 (46.15)	8 (53.33)		36 (66.67)	32 (60.38)	
- Stable infiltration	7 (53.85)	7 (46.67)		18 (33.33)	21 (39.62)	
- Worsening	0 (0.00)	0 (0.00)		0 (0.00)	0 (0.00)	
- Improvement	0 (0.00)	0 (0.00)		0 (0.00)	0 (0.00)	
Pneumonia presented on Day 0, n (%)			0.743[1]			1.000[2]
- Mild illness	7 (53.85)	9 (60.00)		39 (72.22)	38 (71.70)	
- Pneumonia	6 (46.15)	6 (40.00)		15 (27.78)	15 (28.30)	
Pneumonia presented on Day 5, n (%)			0.705[1]			0.499[1]
- Mild illness	6 (46.15)	8 (53.33)		36 (66.67)	32 (60.38)	
- Pneumonia	7 (53.85)	7 (46.67)		18 (33.33)	21 (39.62)	
Negative PCR Day 5, n (%)	1 (8.33)	1 (6.67)	1.000[2]	8 (14.81)	4 (7.55)	0.234[1]
Negative PCR Day 14, n (%)	10 (76.93)	10 (66.67)	0.549[1]	52 (96.30)	47 (88.68)	0.161[2]
WHO-CPS score on Day 0, n (%)			1.000[2]			1.000[2]
- 0	0 (0.00)	0 (0.00)		0 (0.00)	0 (0.00)	
- 1	0 (0.00)	0 (0.00)		54 (100.00)	53 (100.00)	
- 2	13 (100.00)	15 (100.00)		0 (0.00)	0 (0.00)	
WHO-CPS score on Day 5, n (%)			0.783[2]			0.651[2]
- 0	1 (7.69)	1 (6.67)		6 (11.11)	3 (5.66)	
- 1	10 (76.92)	13 (86.67)		43 (79.63)	45 (84.91)	
- 2	2 (15.38)	1 (6.67)		5 (9.26)	5 (9.43)	
WHO-CPS score on Day 14, n (%)			0.435[2]			0.123[2]
- 0	10 (76.92)	9 (60.00)		50 (92.59)	43 (81.13)	
- 1	3 (23.08)	6 (40.00)		4 (7.41)	8 (15.09)	
- 2	0 (0.00)	0 (0.00)		0 (0.00)	2 (3.77)	

Abbreviatioons; WHO-CPS: World Health Organization Clinical Progression Scale; n: number; PCR: Polymerase Chain Reaction

[1]Pearson chi-square test [2]Fisher's exact test

Table S12 Subgroup analysis of individuals who pneumonia presented on day 5 of sotrovimab and combined COVID-19 convalescent plasma with favipiravir treatment.

	**Pneumonia presented on day 5**			
	**Total**	**Sotrovimab**	**Convalescent Plasma with Favipiravir**	**p-value**
Overall clinical, n (%)				0.616[2]
- Stable	3 (5.56)	2 (7.69)	1 (3.57)	
- Worsening	5 (9.26)	3 (11.54)	2 (7.14)	
- Improvement	46 (85.19)	21 (80.77)	25 (89.29)	
Oxygen saturation, mean ± SD	98.82 ± 1.21	98.88 ± 1.17	98.77 ± 1.27	0.836[4]
WHO-CPS score, n (%)				0.200[2]
- 0	1 (1.85)	1 (3.85)	0 (0.00)	
- 1	45 (83.33)	20 (76.92)	25 (89.29)	
- 2	7 (12.96)	0 (0.00)	0 (0.00)	
- 3	0 (0.00)	0 (0.00)	0 (0.00)	
- 4	0 (0.00)	5 (19.23)	2 (7.14)	
- 5	1 (1.85)	0 (0.00)	1 (3.57)	
Progression of pneumonia on chest CT, n (%)				0.150[1]
- Stable pneumonia	41 (75.93)	22 (84.62)	19 (67.86)	
- Progression	13 (24.07)	4 (15.38)	9 (32.14)	
CT severity score day 5, mean ± SD	2.26 ± 2.06	2.18 ± 1.53	2.33 ± 2.48	0.944[4]
SARS-CoV-2 PCR E-gene Day 5, mean ± SD	28.71 ± 4.70	28.49 ± 4.72	28.91 ± 4.76	0.746[3]
SARS-CoV-2 PCR ORF-1 a/b, mean ± SD	27.69 ± 3.94	27.52 ± 3.89	27.84 ± 4.05	0.771[3]
ALT, mean ± SD	24.83 ± 21.41	21.52 ± 12.85	27.9 ± 26.96	0.628[4]
Cr Day 5, mean ± SD	0.93 ± 0.78	1.03 ± 0.84	0.93 ± 0.78	0.808[4]
IL-1α Day 5, mean ± SD	0.30 ± 0.75	0.16 ± 0.24	0.42 ± 0.99	0.419[4]
IL-1β Day 5, mean ± SD	3.39 ± 11.09	0.71 ± 0.40	5.53 ± 14.85	0.448[4]
IL-6 Day 5, mean ± SD	32.19 ± 120.63	2.22 ± 1.58	56.17 ± 161.39	0.213[4]
IL-10 Day 5, mean ± SD	37.82 ± 156.77	0.53 ± 0.35	67.66 ± 210.25	0.594[4]
IFN-γ Day 5, mean ± SD	0.58 ± 1.12	0.59 ± 0.99	0.57 ± 1.27	0.686[4]
TNF-α Day 5, mean ± SD	12.38 ± 24.09	4.39 ± 1.62	18.77 ± 31.49	0.424[4]
MCP-1 Day 5, mean ± SD	143.79 ± 67.04	143.29 ± 71.47	144.18 ± 67.20	0.929[4]

Abbreviatioons; WHO-CPS: World Health Organization Clinical Progression Scale; n: number; PCR: Polymerase Chain Reaction; ALT: alanin aminotransferase; Cr: creatinine; IL: interleukin; IFN: interferon; TNF: Tunor Necrotic Factor: MCP: Monocyte Chemoattractant Protein; CT: Computed tomography

[1]Pearson chi-square test, [2]Fisher's exact test, [3]Independent t-test, [4]Mann-Whitney U test

Figure S1 Cytokines response after sotrovimab versus combined COVID-19 convalescent plasma with favipiravir treatment

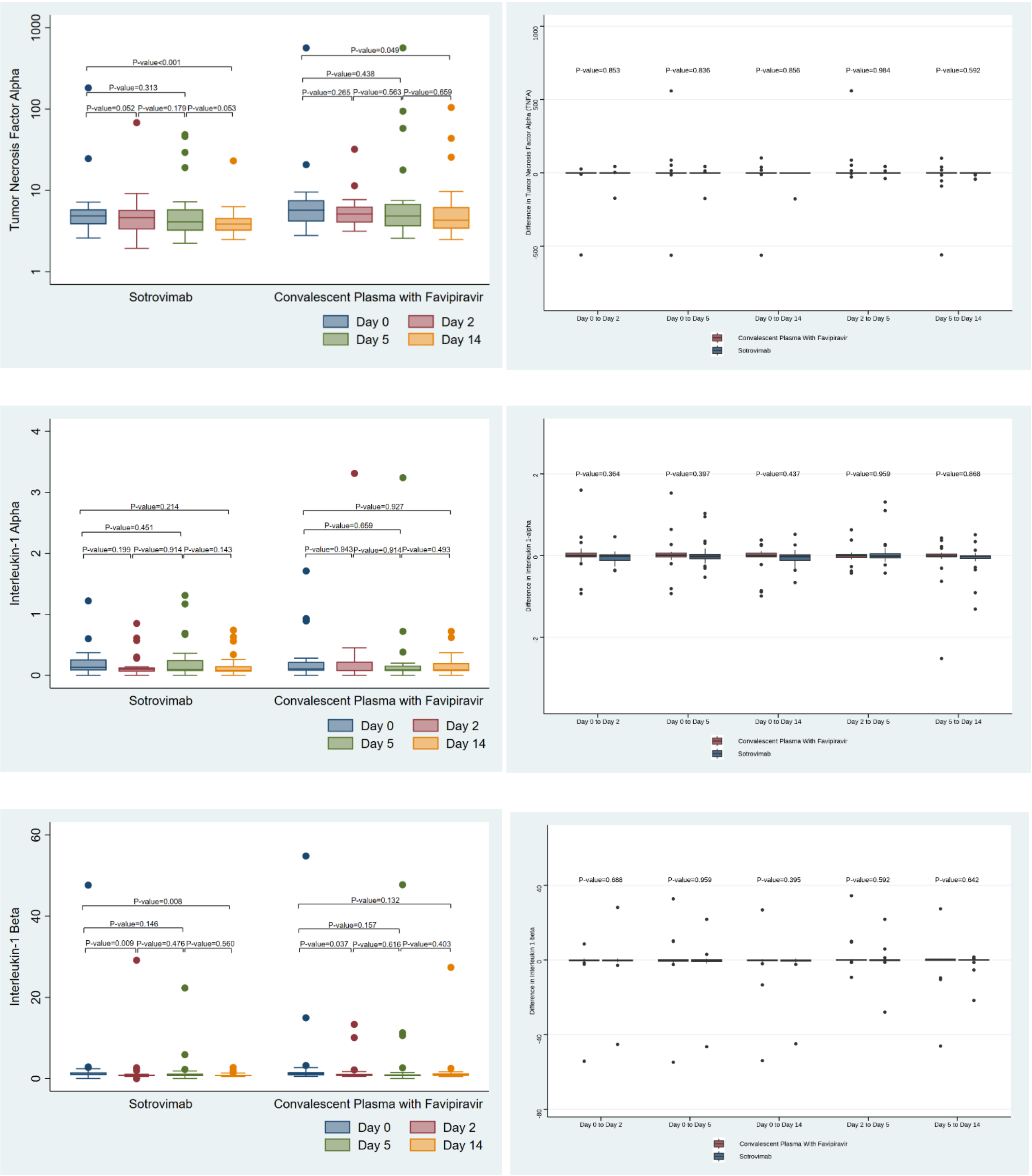

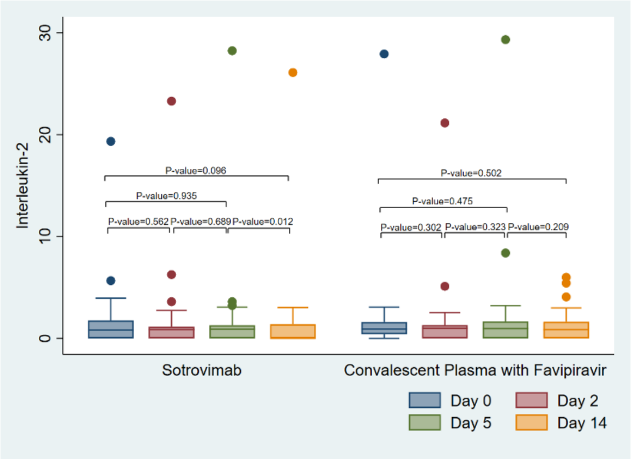
Interleukin-2
P-value=0.096
P-value=0.935
P-value=0.562
P-value=0.689
P-value=0.012
P-value=0.502
P-value=0.475
P-value=0.302
P-value=0.323
P-value=0.209
Sotrovimab
Convalescent Plasma with Favipiravir
Day 0
Day 2
Day 5
Day 14

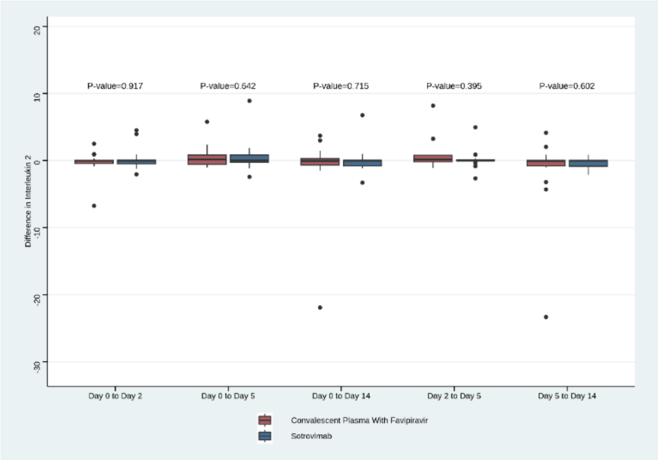
Difference in Interleukin 2
P-value=0.917
P-value=0.642
P-value=0.715
P-value=0.395
P-value=0.602
Day 0 to Day 2
Day 0 to Day 5
Day 0 to Day 14
Day 2 to Day 5
Day 5 to Day 14
Convalescent Plasma With Favipiravir
Sotrovimab

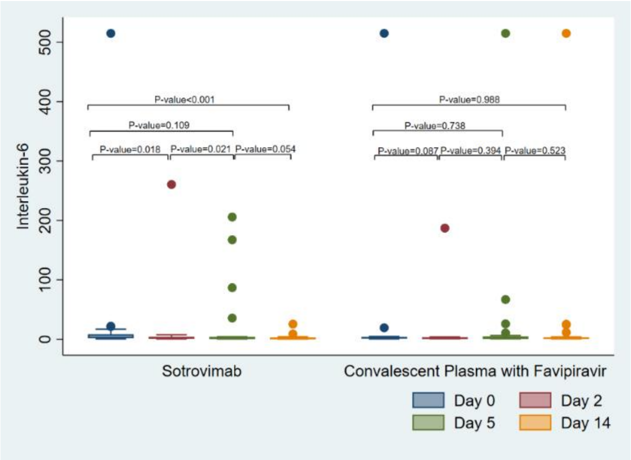
Interleukin-6
P-value<0.001
P-value=0.109
P-value=0.018
P-value=0.021
P-value=0.054
P-value=0.988
P-value=0.738
P-value=0.087
P-value=0.394
P-value=0.523
Sotrovimab
Convalescent Plasma with Favipiravir
Day 0
Day 2
Day 5
Day 14

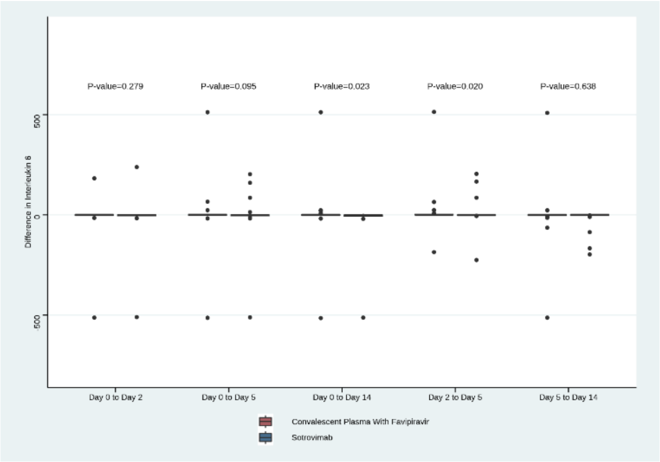
Difference in Interleukin 6
P-value=0.279
P-value=0.095
P-value=0.023
P-value=0.020
P-value=0.638
Day 0 to Day 2
Day 0 to Day 5
Day 0 to Day 14
Day 2 to Day 5
Day 5 to Day 14
Convalescent Plasma With Favipiravir
Sotrovimab

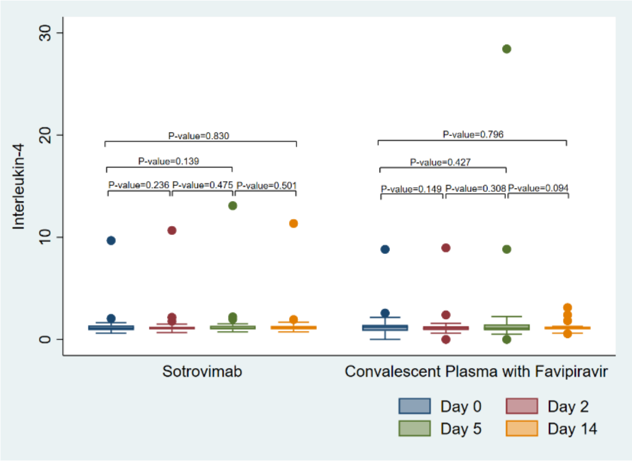
Interleukin-4
P-value=0.830
P-value=0.139
P-value=0.236
P-value=0.475
P-value=0.501
P-value=0.796
P-value=0.427
P-value=0.149
P-value=0.308
P-value=0.094
Sotrovimab
Convalescent Plasma with Favipiravir
Day 0
Day 2
Day 5
Day 14

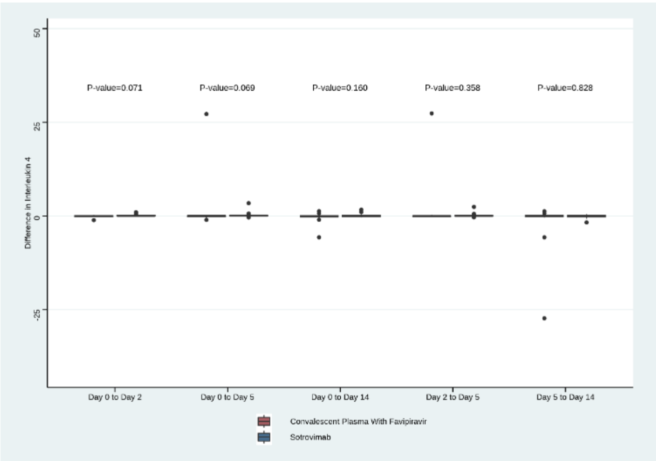
Difference in Interleukin 4
P-value=0.071
P-value=0.069
P-value=0.160
P-value=0.358
P-value=0.828
Day 0 to Day 2
Day 0 to Day 5
Day 0 to Day 14
Day 2 to Day 5
Day 5 to Day 14
Convalescent Plasma With Favipiravir
Sotrovimab

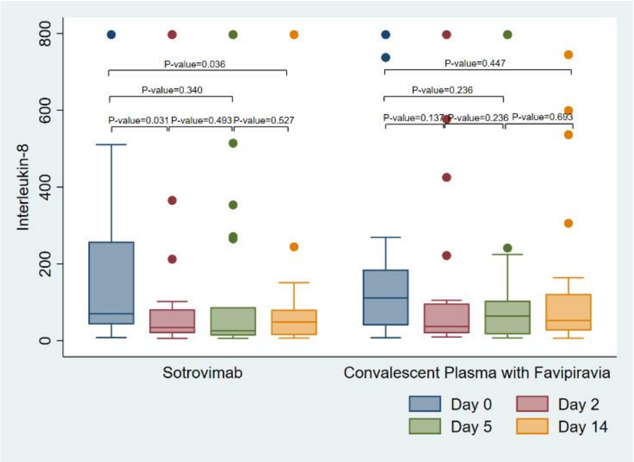

Interleukin-8
P-value=0.036
P-value=0.340
P-value=0.031 P-value=0.493 P-value=0.527
P-value=0.447
P-value=0.236
P-value=0.137 P-value=0.236 P-value=0.693
Sotrovimab
Convalescent Plasma with Favipiravia
Day 0 Day 2
Day 5 Day 14

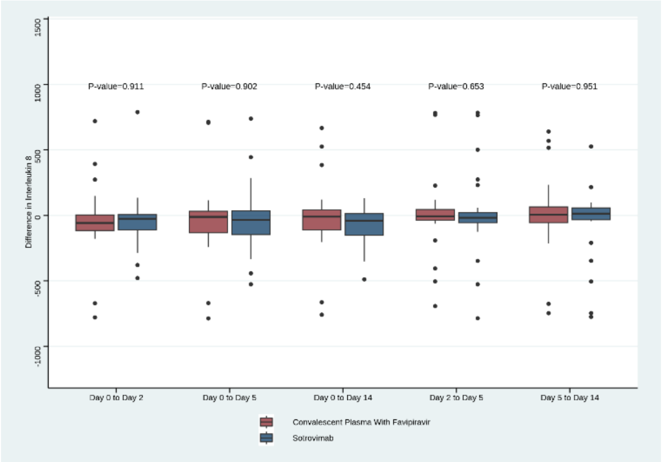

Difference in Interleukin 8
P-value=0.911
P-value=0.902
P-value=0.454
P-value=0.653
P-value=0.951
Day 0 to Day 2
Day 0 to Day 5
Day 0 to Day 14
Day 2 to Day 5
Day 5 to Day 14
Convalescent Plasma With Favipiravir
Sotrovimab

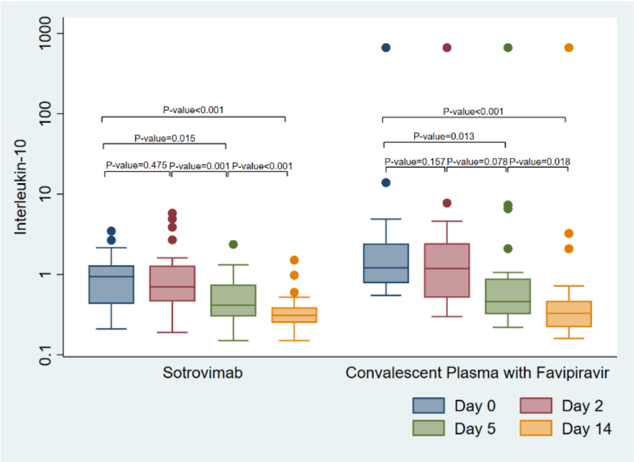

Interleukin-10
P-value<0.001
P-value=0.015
P-value=0.475 P-value=0.001 P-value<0.001
P-value<0.001
P-value=0.013
P-value=0.157 P-value=0.078 P-value=0.018
Sotrovimab
Convalescent Plasma with Favipiravir
Day 0 Day 2
Day 5 Day 14

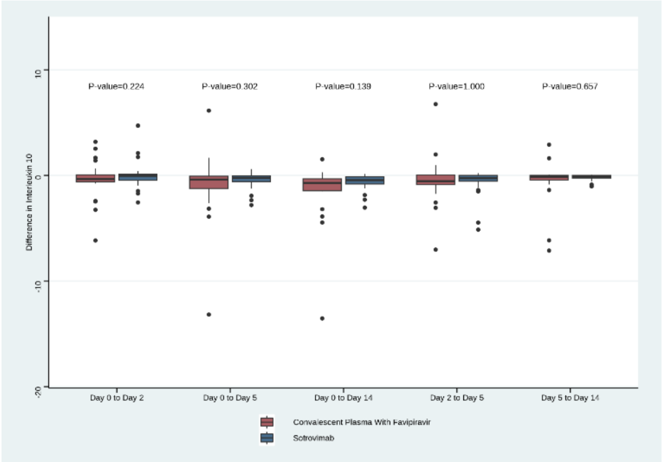

Difference in Interleukin 10
P-value=0.224
P-value=0.302
P-value=0.139
P-value=1.000
P-value=0.657
Day 0 to Day 2
Day 0 to Day 5
Day 0 to Day 14
Day 2 to Day 5
Day 5 to Day 14
Convalescent Plasma With Favipiravir
Sotrovimab

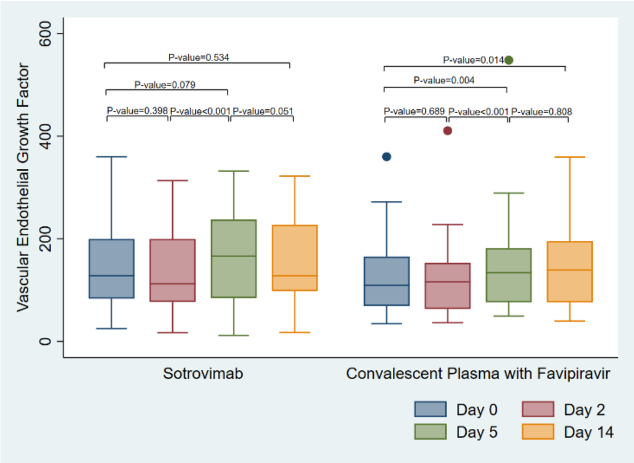

Vascular Endothelial Growth Factor
P-value=0.534
P-value=0.079
P-value=0.398 P-value<0.001 P-value=0.051
P-value=0.014
P-value=0.004
P-value=0.689 P-value<0.001 P-value=0.808
Sotrovimab
Convalescent Plasma with Favipiravir
Day 0 Day 2
Day 5 Day 14

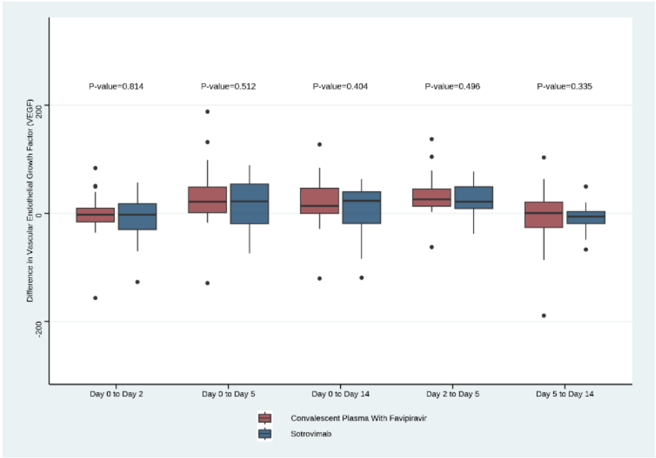

Difference in Vascular Endothelial Growth Factor (VEGF)
P-value=0.814
P-value=0.512
P-value=0.404
P-value=0.496
P-value=0.335
Day 0 to Day 2
Day 0 to Day 5
Day 0 to Day 14
Day 2 to Day 5
Day 5 to Day 14
Convalescent Plasma With Favipiravir
Sotrovimab

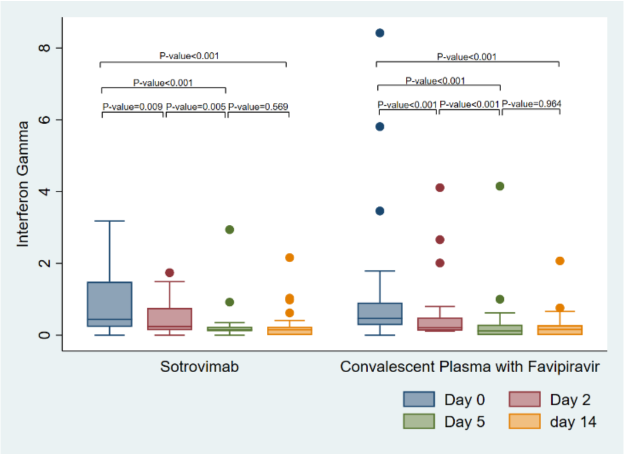

Interferon Gamma
0
2
4
6
8
P-value<0.001
P-value<0.001
P-value=0.009
P-value=0.005
P-value=0.569
P-value<0.001
P-value<0.001
P-value<0.001
P-value<0.001
P-value=0.964
Sotrovimab
Convalescent Plasma with Favipiravir
Day 0
Day 2
Day 5
day 14

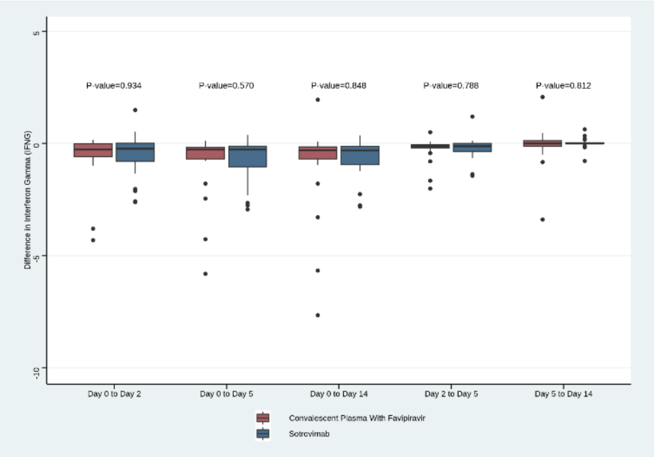

Difference in Interferon Gamma (IFNG)
5
0
-5
-10
P-value=0.934
P-value=0.570
P-value=0.848
P-value=0.788
P-value=0.812
Day 0 to Day 2
Day 0 to Day 5
Day 0 to Day 14
Day 2 to Day 5
Day 5 to Day 14
Convalescent Plasma With Favipiravir
Sotrovimab

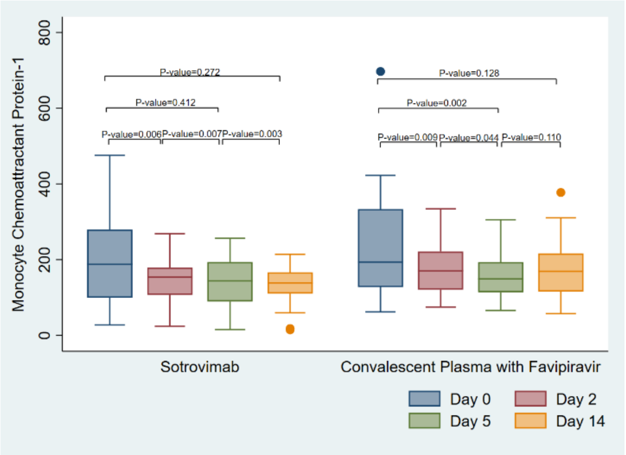

Monocyte Chemoattractant Protein-1
0
200
400
600
800
P-value=0.272
P-value=0.412
P-value=0.006
P-value=0.007
P-value=0.003
P-value=0.128
P-value=0.002
P-value=0.009
P-value=0.044
P-value=0.110
Sotrovimab
Convalescent Plasma with Favipiravir
Day 0
Day 2
Day 5
Day 14

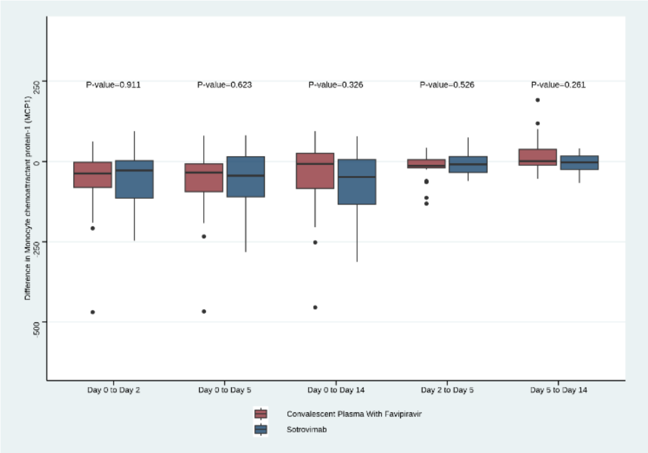

Difference in Monocyte chemoattractant protein-1 (MCP1)
250
0
-250
-500
P-value=0.911
P-value=0.623
P-value=0.326
P-value=0.526
P-value=0.261
Day 0 to Day 2
Day 0 to Day 5
Day 0 to Day 14
Day 2 to Day 5
Day 5 to Day 14
Convalescent Plasma With Favipiravir
Sotrovimab

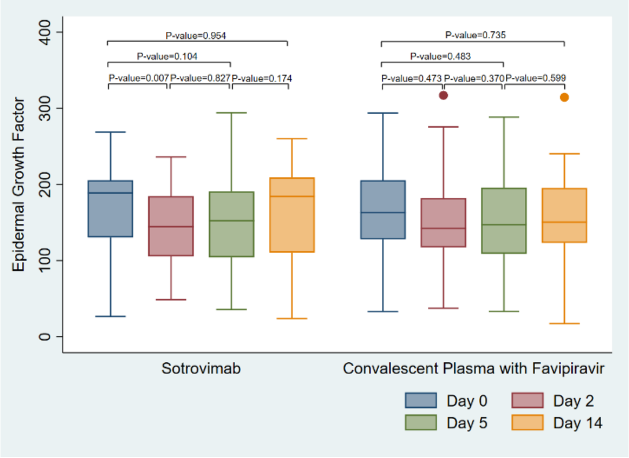

Epidermal Growth Factor
0
100
200
300
400
P-value=0.954
P-value=0.104
P-value=0.007
P-value=0.827
P-value=0.174
P-value=0.735
P-value=0.483
P-value=0.473
P-value=0.370
P-value=0.599
Sotrovimab
Convalescent Plasma with Favipiravir
Day 0
Day 2
Day 5
Day 14

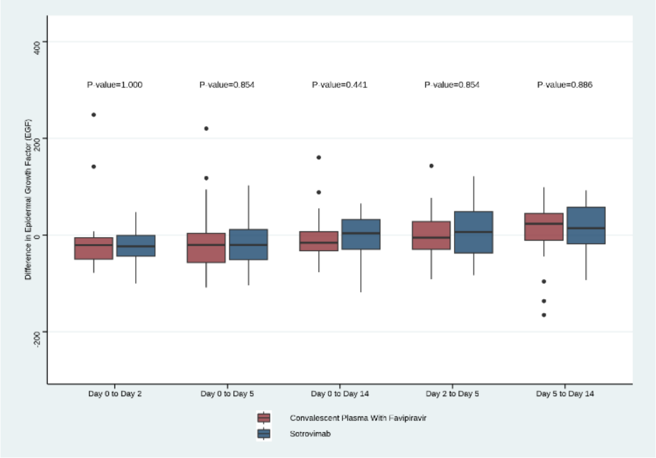

Difference in Epidermal Growth Factor (EGF)
400
200
0
-200
P-value=1.000
P-value=0.854
P-value=0.441
P-value=0.854
P-value=0.886
Day 0 to Day 2
Day 0 to Day 5
Day 0 to Day 14
Day 2 to Day 5
Day 5 to Day 14
Convalescent Plasma With Favipiravir
Sotrovimab

Figure S2 Nucleocapsid antibody response after sotrovimab versus combined COVID-19 convalescent plasma with favipiravir treatment

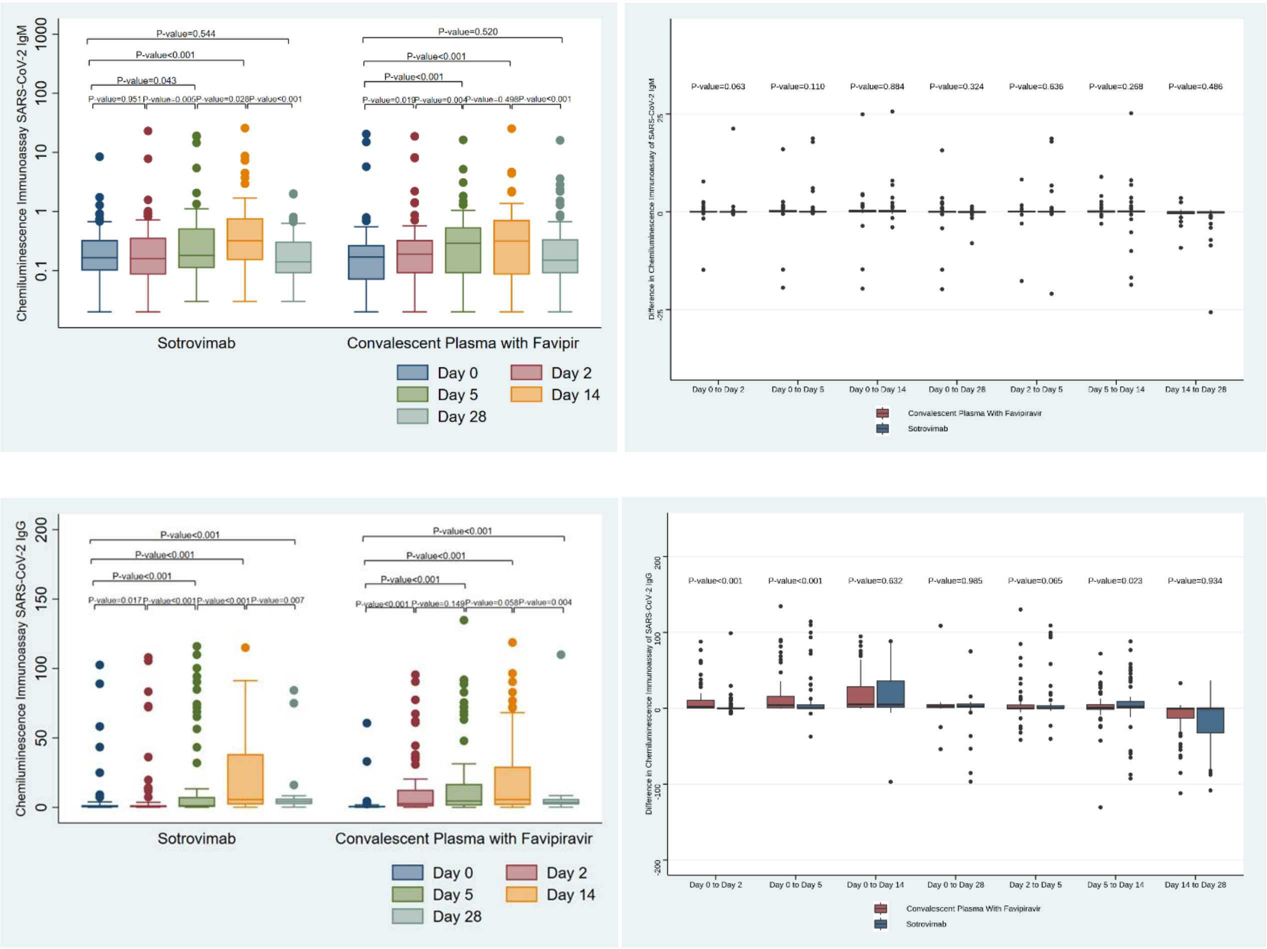

Figure S3 Anti-RBD spike IgG and surrogated virus neutralizing test (sVNT) change after sotrovimab versus combined COVID-19 convalescent plasma with favipiravir treatment

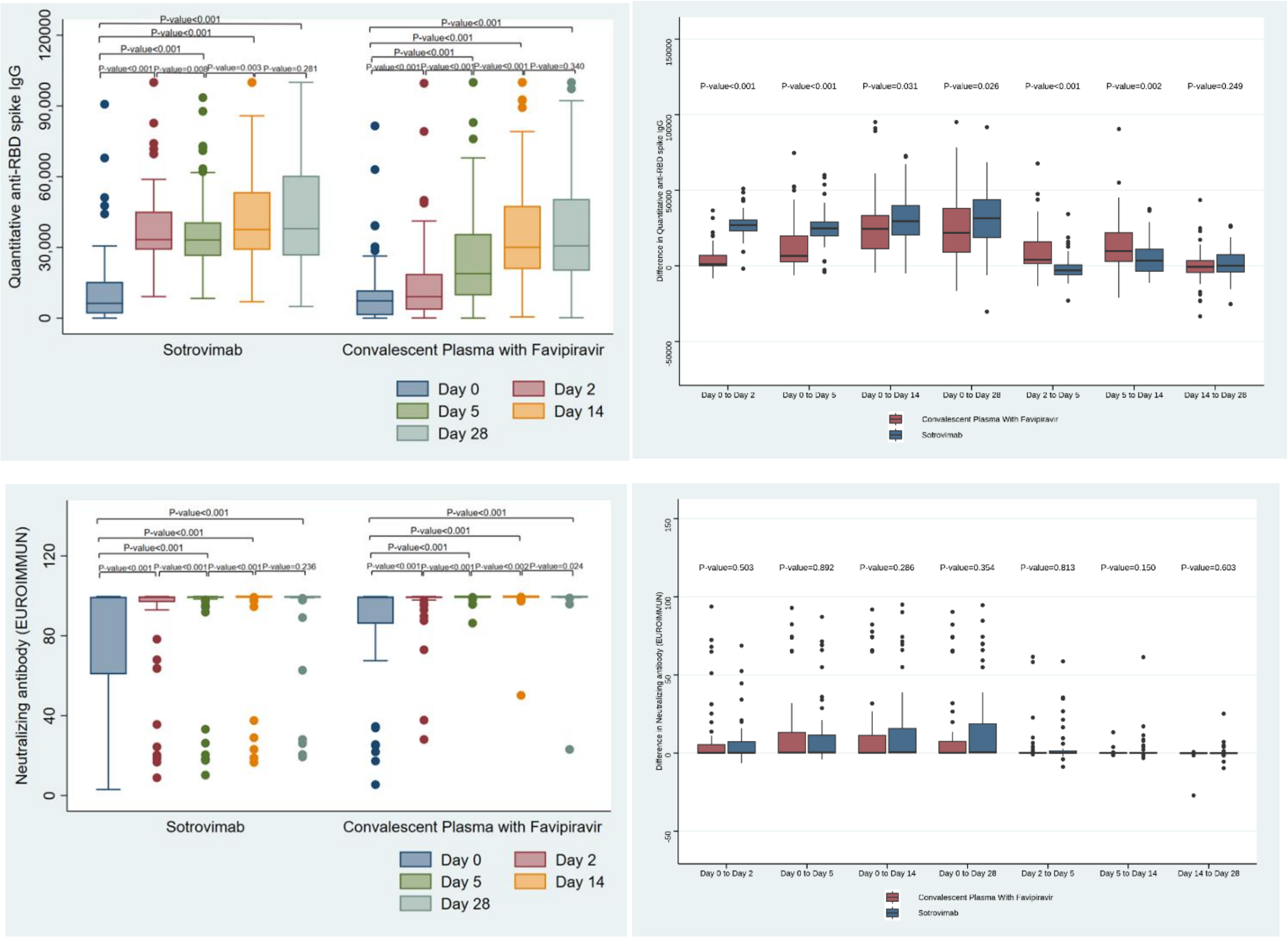

Figure S4 Chest computed tomography severity index score change between day 0 and 5 of sotrovimab versus combined COVID-19 convalescent plasma with favipiravir treatment

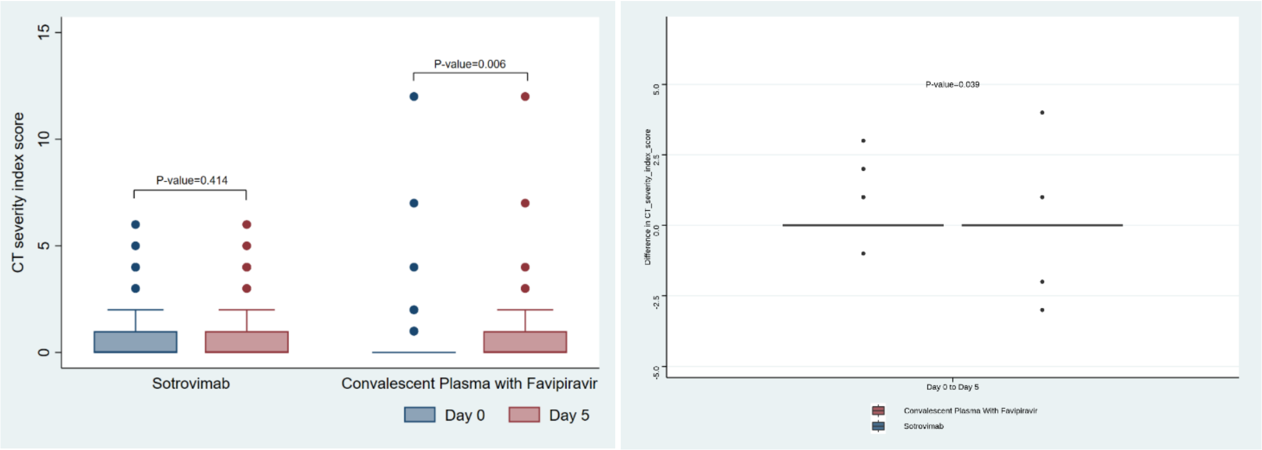

Figure S5 Hepatic enzymes change between sotrovimab versus combined COVID-19 convalescent plasma with favipiravir treatment

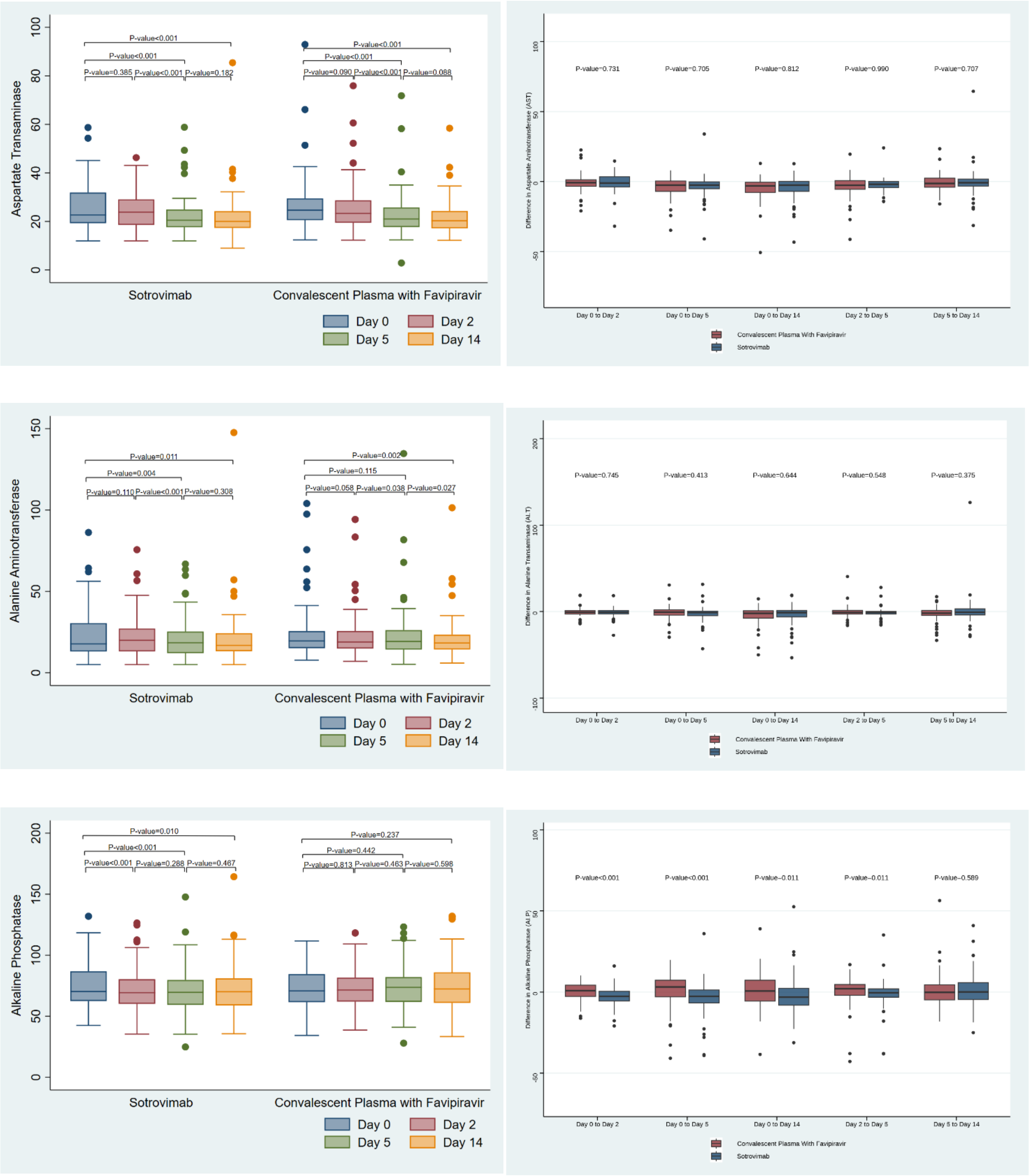

Figure S6 Inflammatory markers change between days 0 and 5 of sotrovimab versus combined COVID-19 convalescent plasma with favipiravir treatment

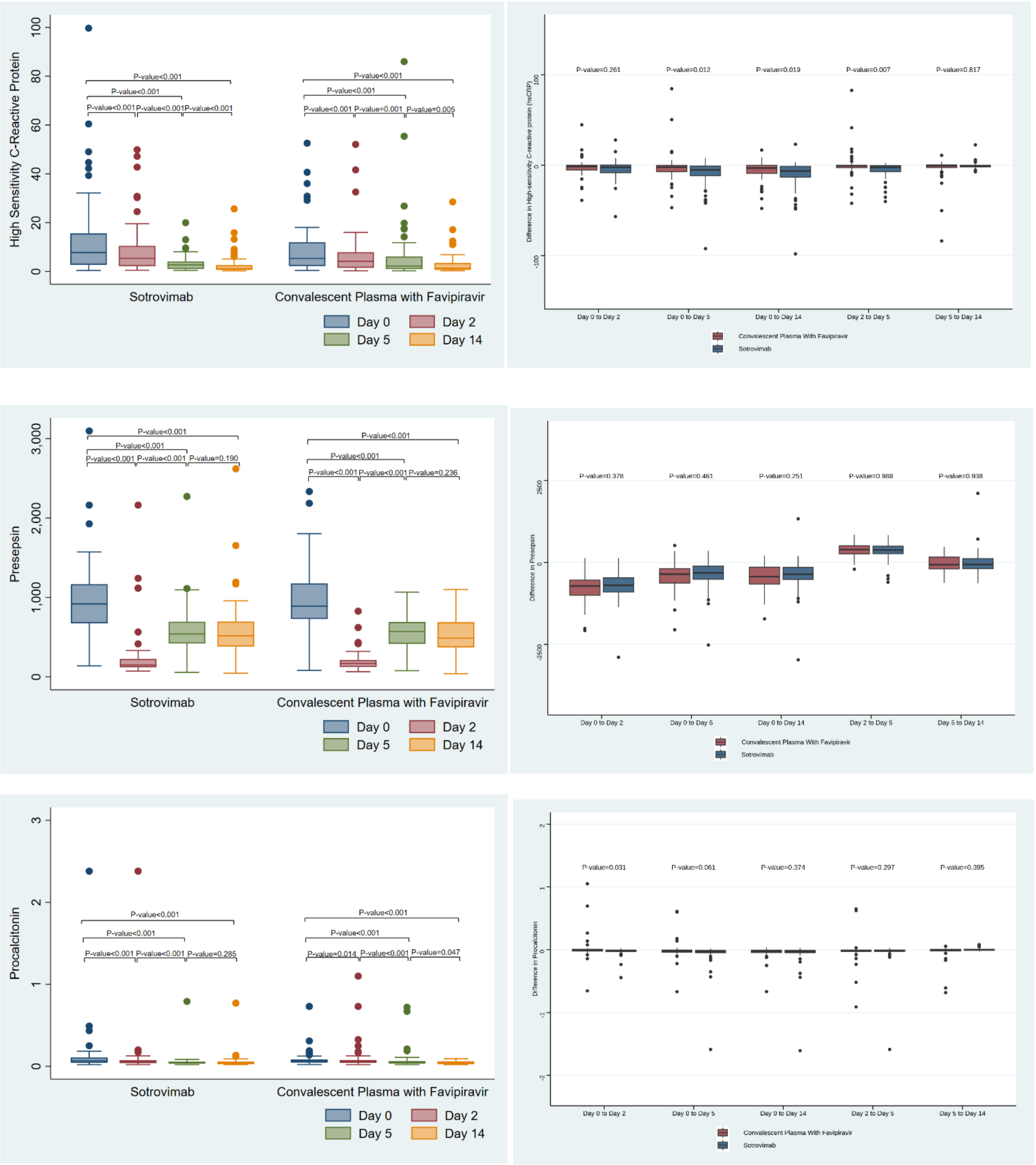

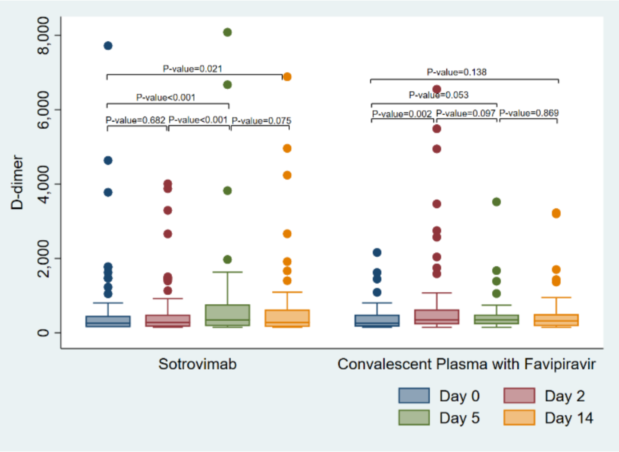

D-dimer
0
2,000
4,000
6,000
8,000
P-value=0.021
P-value<0.001
P-value=0.682 P-value<0.001 P-value=0.075
P-value=0.138
P-value=0.053
P-value=0.002 P-value=0.097 P-value=0.869
Sotrovimab
Convalescent Plasma with Favipiravir
Day 0
Day 2
Day 5
Day 14

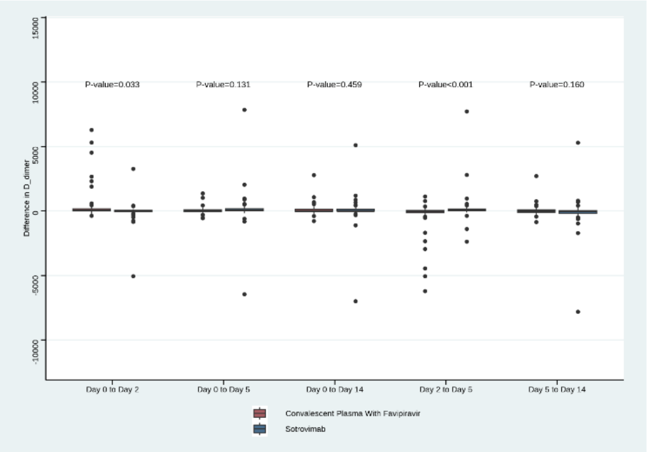

Difference in D_dimer
P-value=0.033
P-value=0.131
P-value=0.459
P-value<0.001
P-value=0.160
Day 0 to Day 2
Day 0 to Day 5
Day 0 to Day 14
Day 2 to Day 5
Day 5 to Day 14
Convalescent Plasma With Favipiravir
Sotrovimab

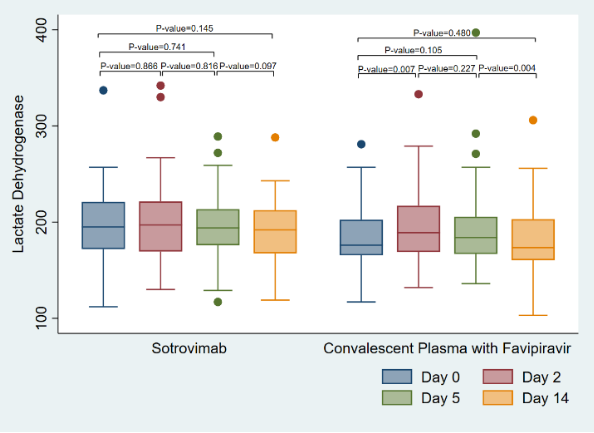

Lactate Dehydrogenase
100
200
300
400
P-value=0.145
P-value=0.741
P-value=0.866 P-value=0.816 P-value=0.097
P-value=0.480
P-value=0.105
P-value=0.007 P-value=0.227 P-value=0.004
Sotrovimab
Convalescent Plasma with Favipiravir
Day 0
Day 2
Day 5
Day 14

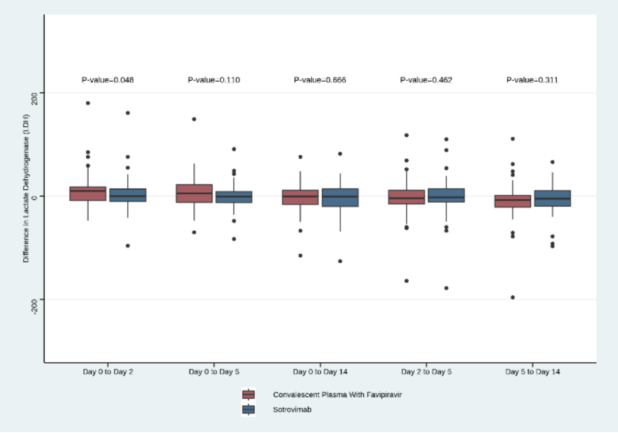

Difference in Lactate Dehydrogenase (LDH)
P-value=0.048
P-value=0.110
P-value=0.666
P-value=0.462
P-value=0.311
Day 0 to Day 2
Day 0 to Day 5
Day 0 to Day 14
Day 2 to Day 5
Day 5 to Day 14
Convalescent Plasma With Favipiravir
Sotrovimab

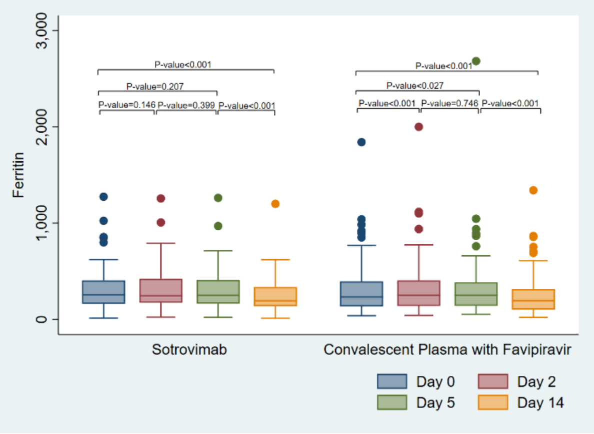

Ferritin
0
1,000
2,000
3,000
P-value<0.001
P-value=0.207
P-value=0.146 P-value=0.399 P-value<0.001
P-value<0.001
P-value=0.027
P-value<0.001 P-value=0.746 P-value<0.001
Sotrovimab
Convalescent Plasma with Favipiravir
Day 0
Day 2
Day 5
Day 14

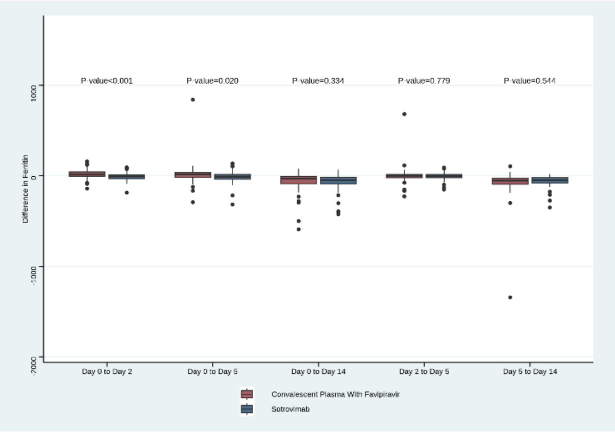

Difference in Ferritin
P-value<0.001
P-value=0.020
P-value=0.334
P-value=0.779
P-value=0.544
Day 0 to Day 2
Day 0 to Day 5
Day 0 to Day 14
Day 2 to Day 5
Day 5 to Day 14
Convalescent Plasma With Favipiravir
Sotrovimab

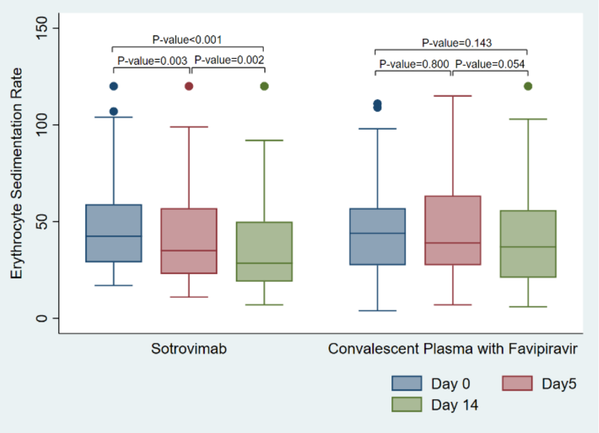

Erythrocyte Sedimentation Rate
0
50
100
150
P-value<0.001
P-value=0.003 P-value=0.002
P-value=0.143
P-value=0.800 P-value=0.054
Sotrovimab
Convalescent Plasma with Favipiravir
Day 0
Day5
Day 14

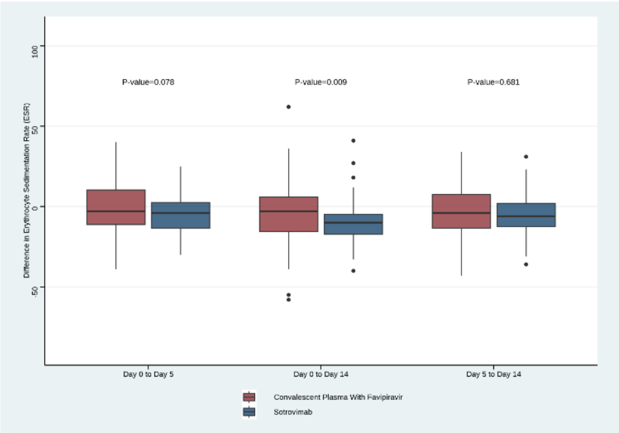

Difference in Erythrocyte Sedimentation Rate (ESR)
P-value=0.078
P-value=0.009
P-value=0.681
Day 0 to Day 5
Day 0 to Day 14
Day 5 to Day 14
Convalescent Plasma With Favipiravir
Sotrovimab

Figure S7 Uric acid level change between sotrovimab versus combined COVID-19 convalescent plasma with favipiravir treatment

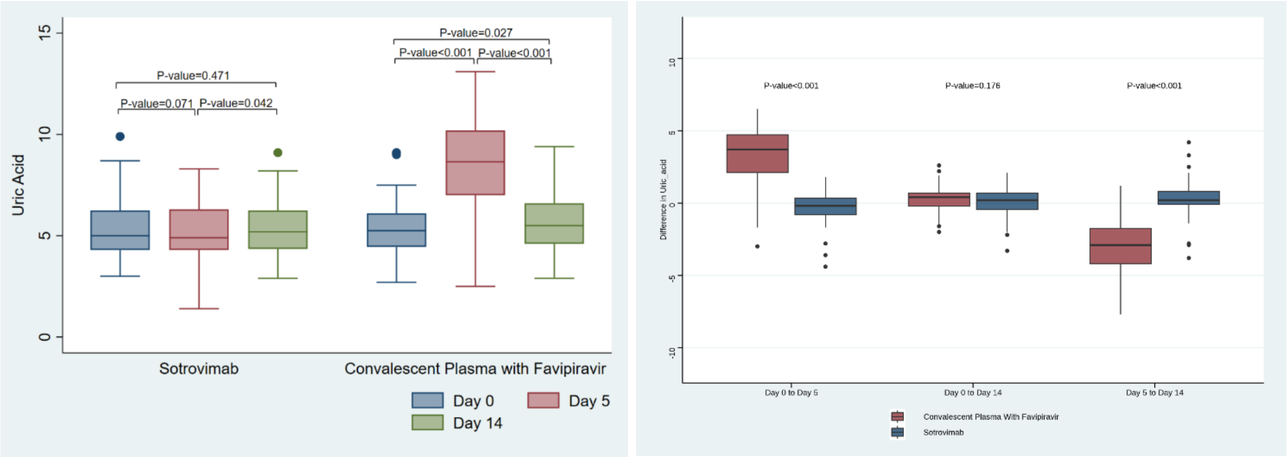

Figure S8 Cycle threshold value from SARS-CoV-2 PCR change after sotrovimab versus combined COVID-19 convalescent plasma with favipiravir treatment.

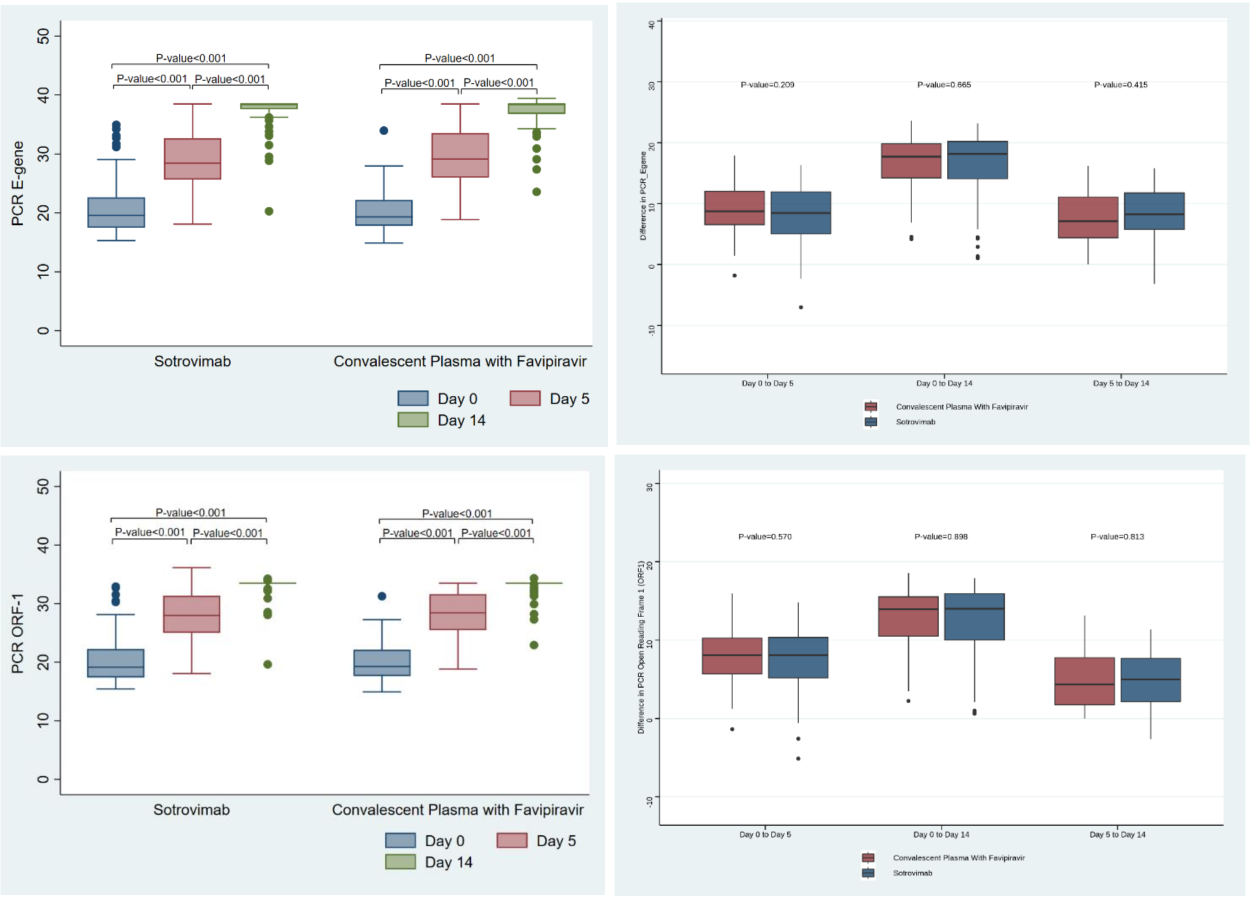

Figure S9 Subgroup analysis of Quantitative anti-RBD spike IgG antibody response among unvaccinated patients received sotrovimab versus combined COVID-19 convalescent plasma with favipiravir treatment.

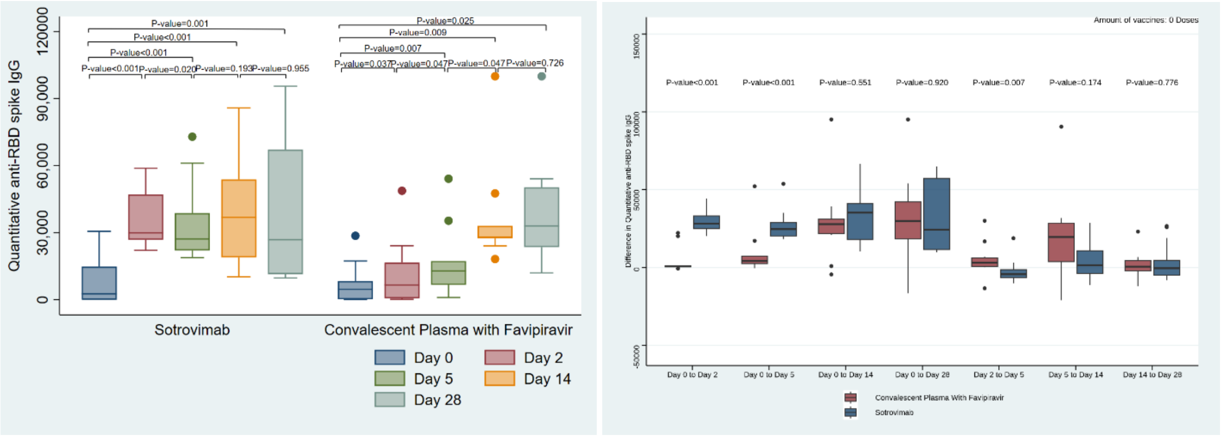

Figure S10 Subgroup analysis of Quantitative anti-RBD spike IgG antibody response among those who received fully more than 2 doses of vaccination before sotrovimab versus combined COVID-19 convalescent plasma with favipiravir treatment

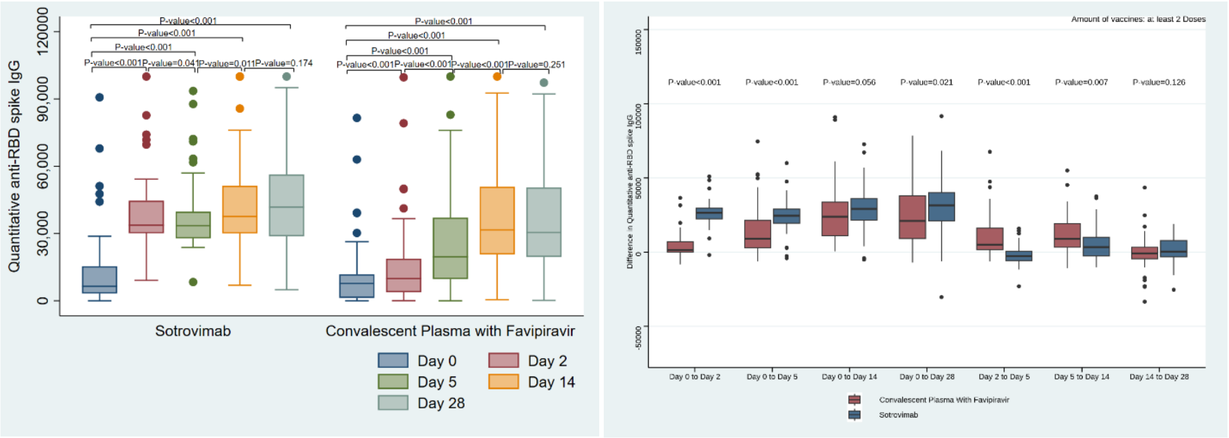

Figure S11 Chest CT severity index score in sotrovimab versus combined COVID-19 convalescent plasma with favipiravir treatment among unvaccinated and at least 2-dose vaccination.

Chest CT severity index score in the unvaccinated group

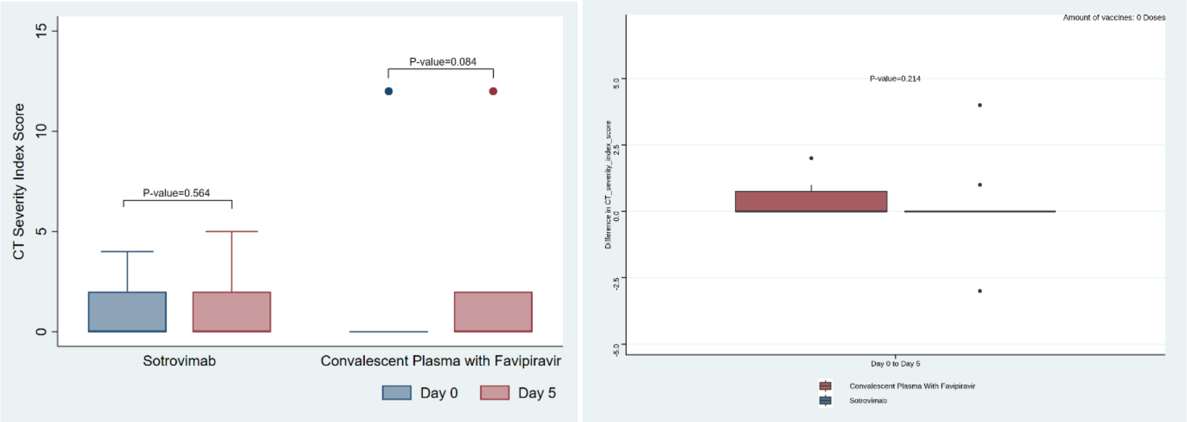

Chest CT severity index score in at least 2 doses vaccinated group.

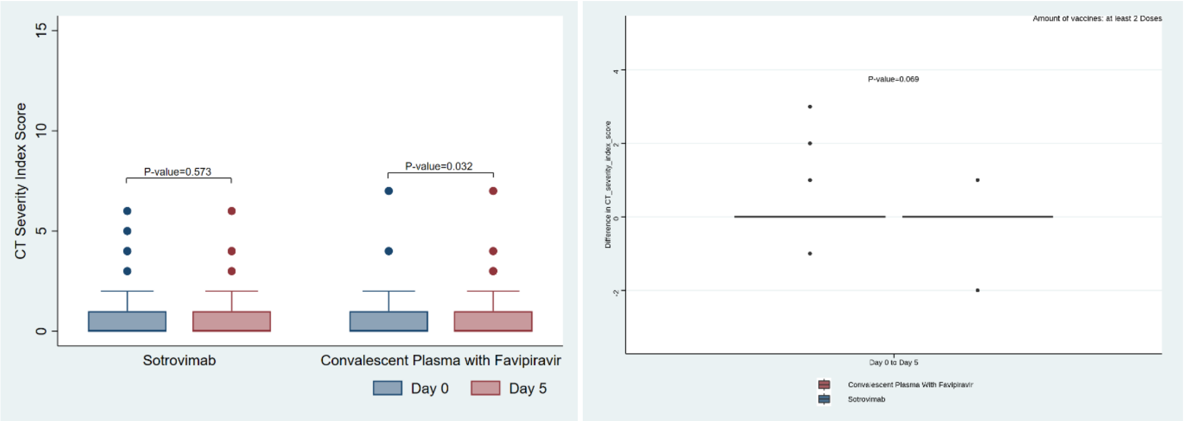

Figure S12 Inflammatory markers change of sotrovimab versus combined COVID-19 convalescent plasma with favipiravir treatment among unvaccinated.

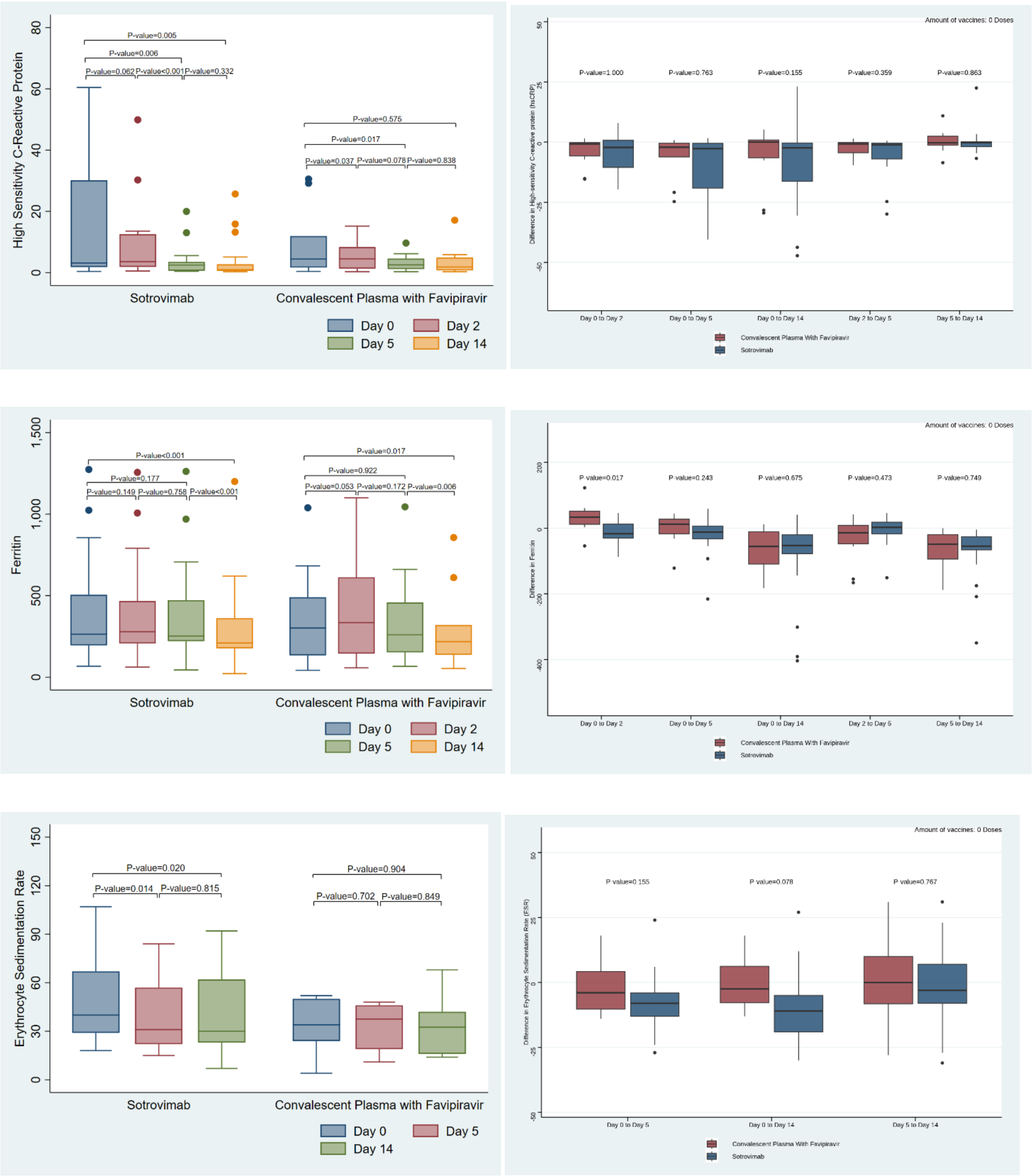

Figure S13 Inflammatory markers change of sotrovimab versus combined COVID-19 convalescent plasma with favipiravir treatment among those who received at least 2-dose vaccination.

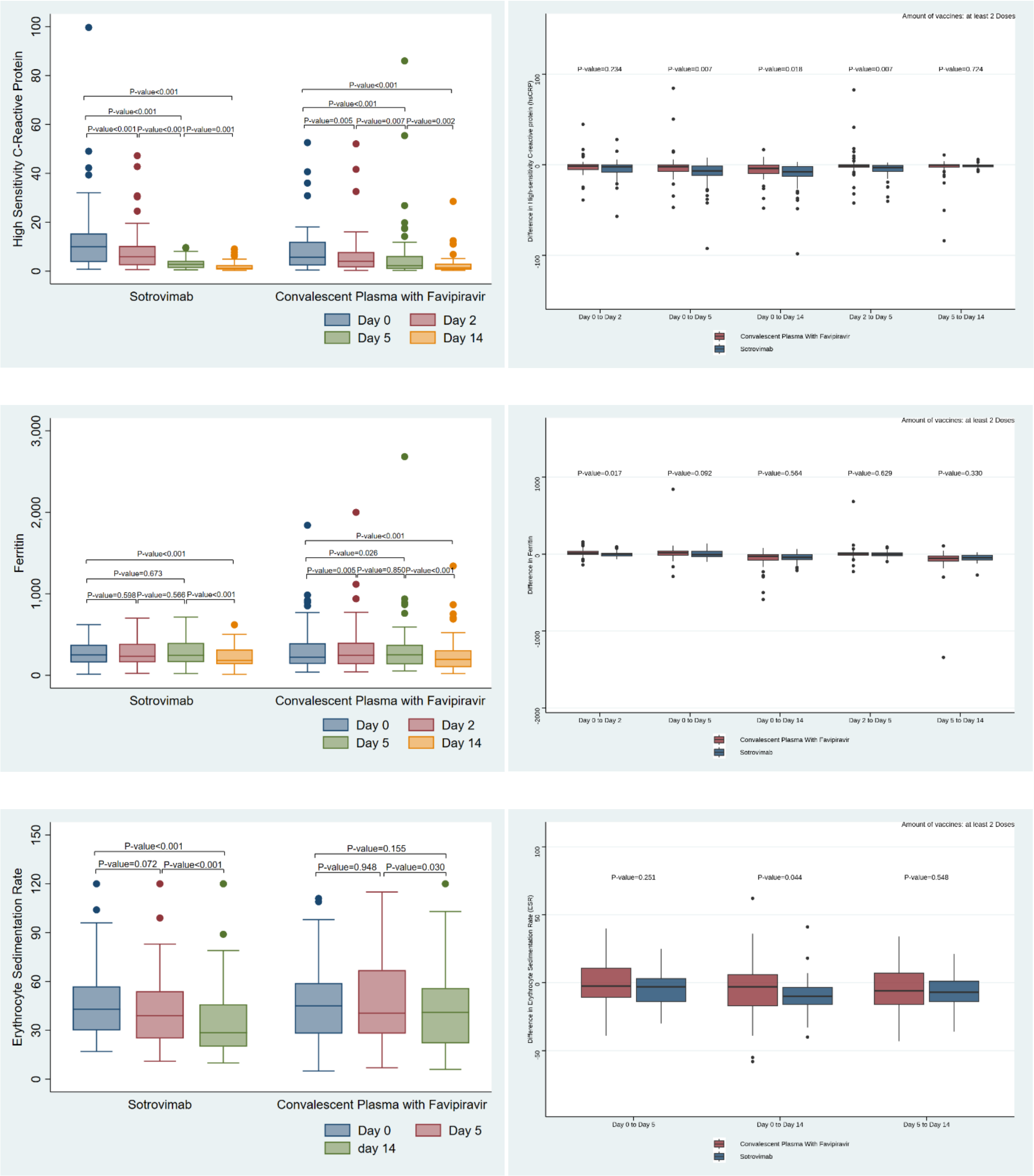

Supplementary Figure S14 The VOC/VOI/VUM relative frequencies over time of SARS-CoV-2 variants from GISAID and monitored variant by Department of Medical Sciences Ministry of Public Health (a.) The overall VOC/VOI/VUM relative frequencies over time in Thailand (b.) Demonstrated VOC consisted of 87% of B.1.1.529, 12% of B.1.617.2 in January 2022 (c.) Demonstrated VOC consisted of 98% of B.1.1.529, BA., 1% of B.1.617.2 in February 2022 (d.) Demonstrated VOC consisted of 99% of B.1.1.529, BA.* in March 2022 (e. and f.) Demonstrated VOC consisted of 100% of B.1.1.529, BA.* in April to May 2022 (g.) Demonstrated VOC consisted of 99.99% of B.1.1.529 and VUM 0.01% of BA.2.75 in June 2022 (https://gisaid.org/hcov-19-variants-dashboard/)*

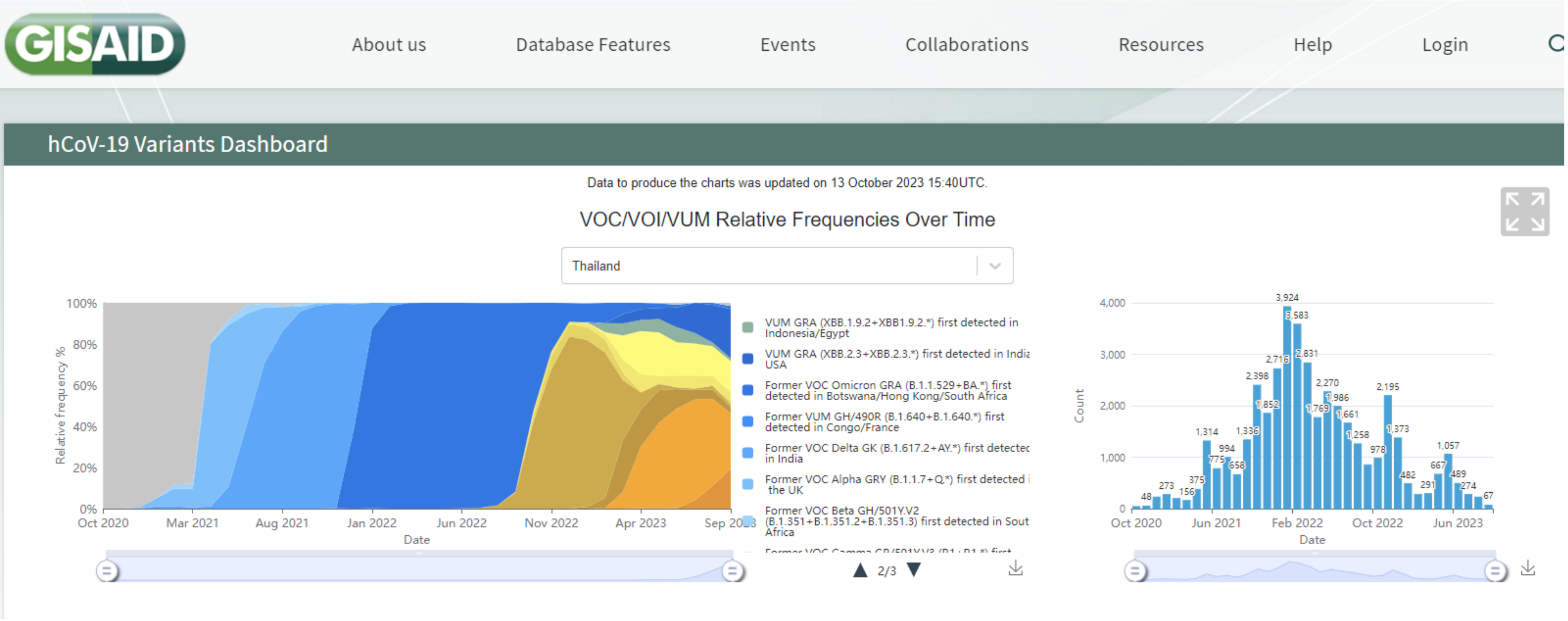

(b.)

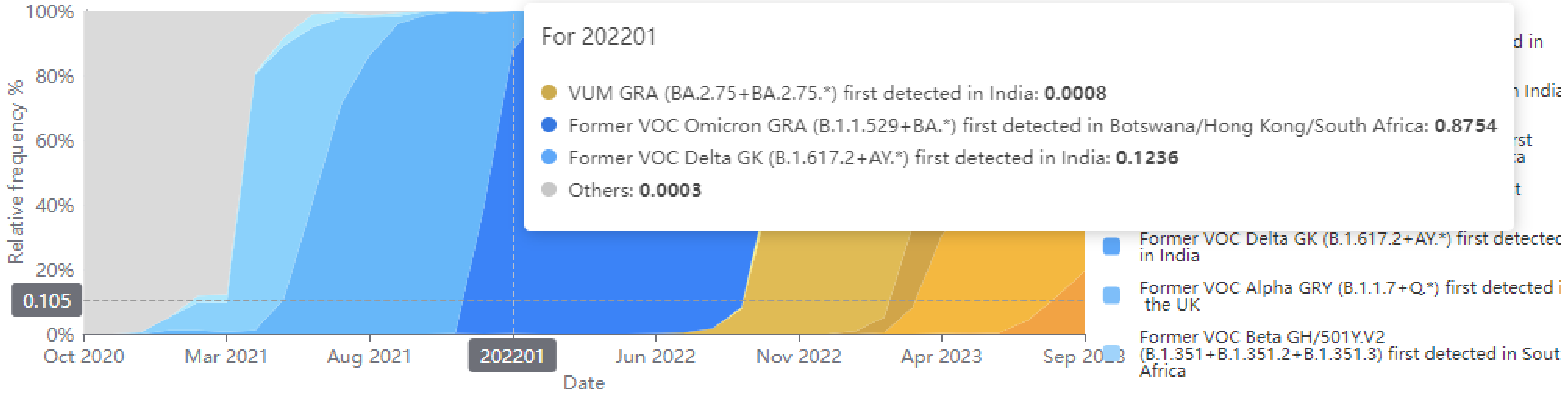

100%
80%
60%
40%
20%
0%
Relative frequency %
0.105
Oct 2020
Mar 2021
Aug 2021
202201
Jun 2022
Nov 2022
Apr 2023
Date
For 202201
VUM GRA (BA.2.75+BA.2.75.*) first detected in India: 0.0008
Former VOC Omicron GRA (B.1.1.529+BA.*) first detected in Botswana/Hong Kong/South Africa: 0.8754
Former VOC Delta GK (B.1.617.2+AY.*) first detected in India: 0.1236
Others: 0.0003
Former VOC Alpha GRY (B.1.1.7+Q.*) first detected i the UK
Former VOC Beta GH/501Y.V2 (B.1.351+B.1.351.2+B.1.351.3) first detected in Sout Africa

(c.)

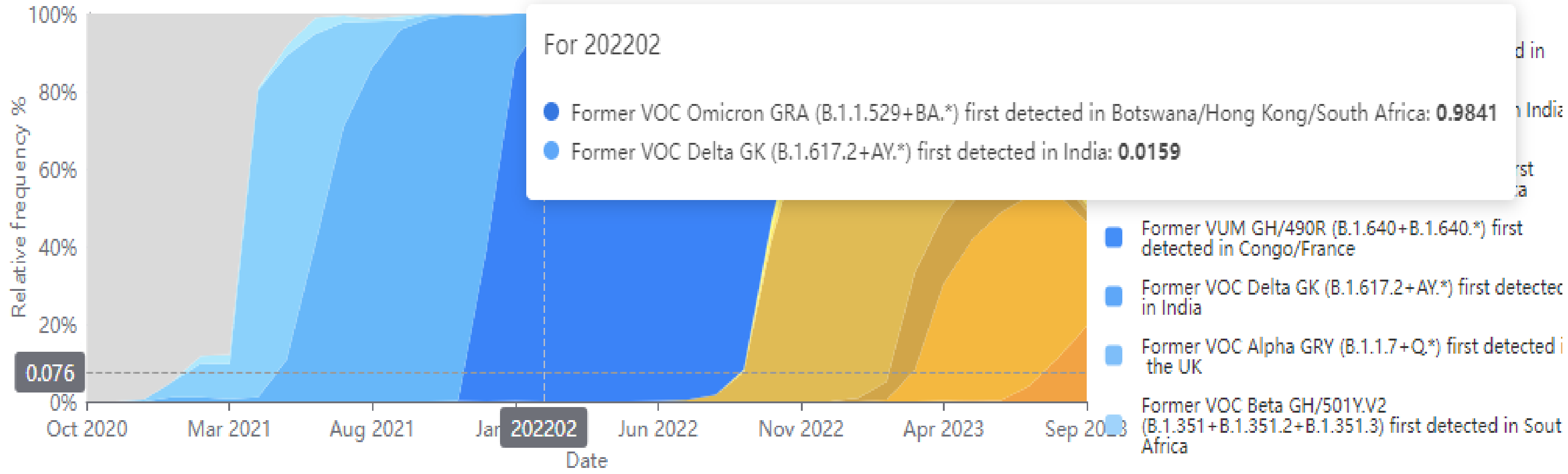

100%
80%
60%
40%
20%
0%
Relative frequency %
0.076
Oct 2020
Mar 2021
Aug 2021
202202
Jun 2022
Nov 2022
Apr 2023
Date
For 202202
Former VOC Omicron GRA (B.1.1.529+BA.*) first detected in Botswana/Hong Kong/South Africa: 0.9841
Former VOC Delta GK (B.1.617.2+AY.*) first detected in India: 0.0159
Former VUM GH/490R (B.1.640+B.1.640.*) first detected in Congo/France
Former VOC Alpha GRY (B.1.1.7+Q.*) first detected i the UK
Former VOC Beta GH/501Y.V2 (B.1.351+B.1.351.2+B.1.351.3) first detected in Sout Africa

(d.)

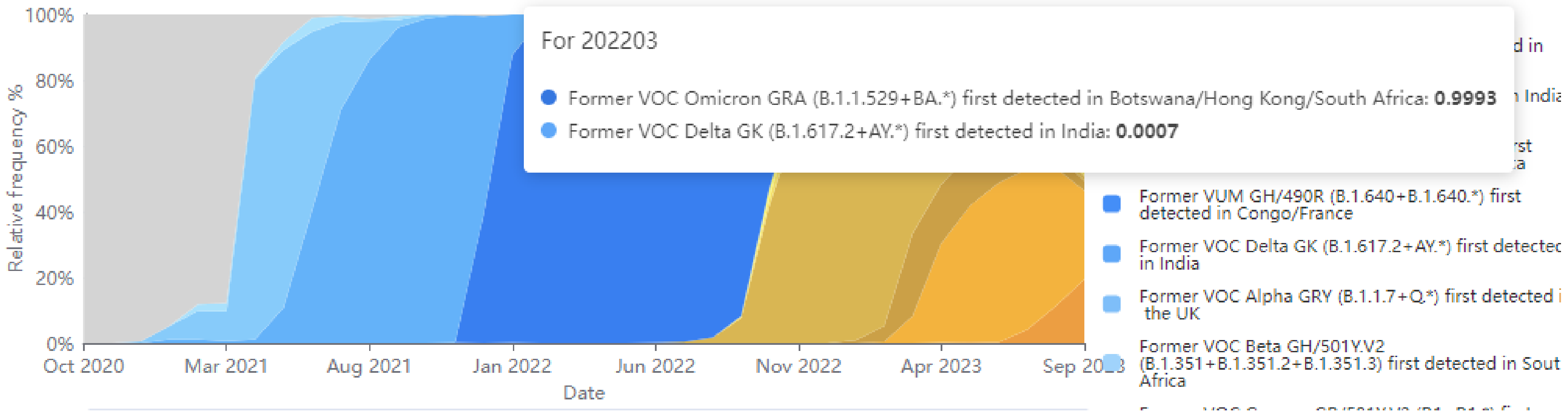
For 202203
Former VOC Omicron GRA (B.1.1.529+BA.*) first detected in Botswana/Hong Kong/South Africa: 0.9993
Former VOC Delta GK (B.1.617.2+AY.*) first detected in India: 0.0007
Relative frequency %
100%
80%
60%
40%
20%
0%
Oct 2020
Mar 2021
Aug 2021
Jan 2022
Jun 2022
Nov 2022
Apr 2023
Date
Former VUM GH/490R (B.1.640+B.1.640.*) first detected in Congo/France
Former VOC Alpha GRY (B.1.1.7+Q.*) first detected i
the UK
Former VOC Beta GH/501Y.V2
(B.1.351+B.1.351.2+B.1.351.3) first detected in Sout
Africa

(e.)

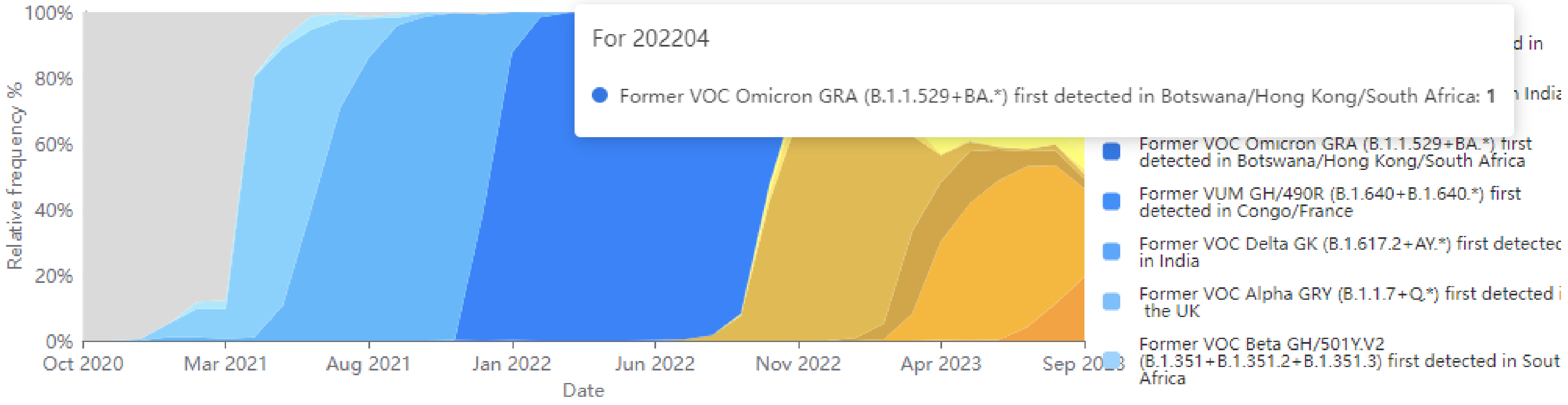
For 202204
Former VOC Omicron GRA (B.1.1.529+BA.*) first detected in Botswana/Hong Kong/South Africa: 1
Relative frequency %
100%
80%
60%
40%
20%
0%
Oct 2020
Mar 2021
Aug 2021
Jan 2022
Jun 2022
Nov 2022
Apr 2023
Date
Former VOC Omicron GRA (B.1.1.529+BA.*) first detected in Botswana/Hong Kong/South Africa
Former VUM GH/490R (B.1.640+B.1.640.*) first detected in Congo/France
Former VOC Delta GK (B.1.617.2+AY.*) first detectec
in India
Former VOC Alpha GRY (B.1.1.7+Q.*) first detected i
the UK
Former VOC Beta GH/501Y.V2
(B.1.351+B.1.351.2+B.1.351.3) first detected in Sout
Africa

(f.)

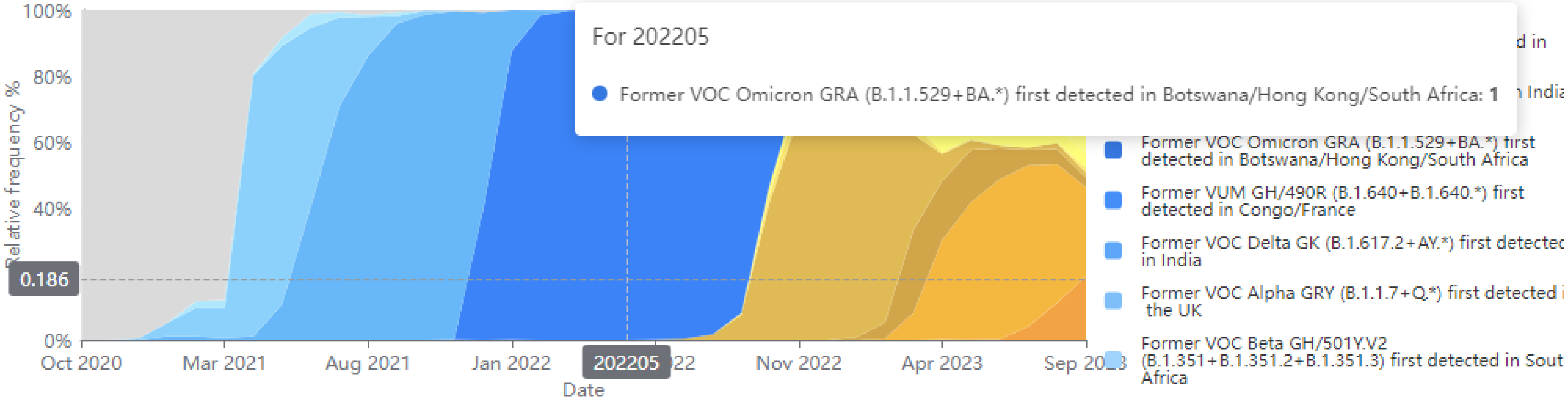
For 202205
Former VOC Omicron GRA (B.1.1.529+BA.*) first detected in Botswana/Hong Kong/South Africa: 1
Relative frequency %
Date
0.186
202205
Former VOC Omicron GRA (B.1.1.529+BA.*) first detected in Botswana/Hong Kong/South Africa
Former VUM GH/490R (B.1.640+B.1.640.*) first detected in Congo/France
Former VOC Delta GK (B.1.617.2+AY.*) first detected in India
Former VOC Alpha GRY (B.1.1.7+Q.*) first detected in the UK
Former VOC Beta GH/501Y.V2 (B.1.351+B.1.351.2+B.1.351.3) first detected in South Africa

(g.)

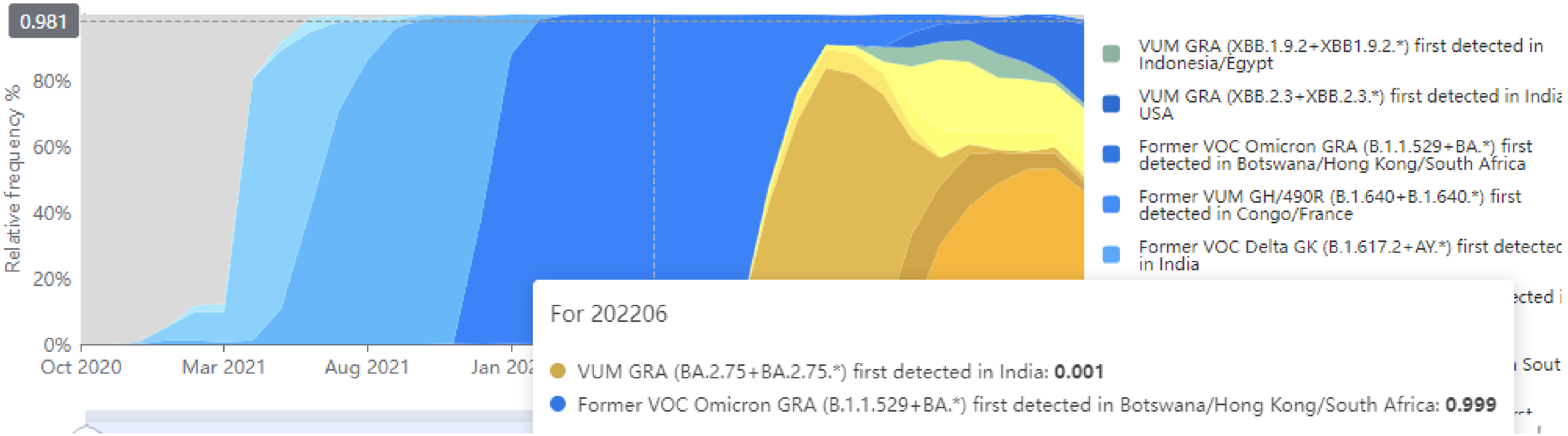
0.981
Relative frequency %
VUM GRA (XBB.1.9.2+XBB1.9.2.*) first detected in Indonesia/Egypt
VUM GRA (XBB.2.3+XBB.2.3.*) first detected in India USA
Former VOC Omicron GRA (B.1.1.529+BA.*) first detected in Botswana/Hong Kong/South Africa
Former VUM GH/490R (B.1.640+B.1.640.*) first detected in Congo/France
Former VOC Delta GK (B.1.617.2+AY.*) first detected in India
For 202206
VUM GRA (BA.2.75+BA.2.75.*) first detected in India: 0.001
Former VOC Omicron GRA (B.1.1.529+BA.*) first detected in Botswana/Hong Kong/South Africa: 0.999
